# Metabolomic Profiling of Malaysian and New Zealand Honey Using Concatenated NMR and HRMS Datasets

**DOI:** 10.3390/metabo12010085

**Published:** 2022-01-17

**Authors:** Yusnaini M. Yusoff, Grainne Abbott, Louise Young, RuAngelie Edrada-Ebel

**Affiliations:** 1 Strathclyde Institute of Pharmacy and Biomedical Sciences, University of Strathclyde, The John Arbuthnott Building, 161 Cathedral Street, Glasgow G4 0RE, UK; grainne.abbott@strath.ac.uk (G.A.); louise.c.young@strath.ac.uk (L.Y.); 2 CADS, Level 8, Vertical Corporate Tower B, Avenue 10, The Vertical, No. 8 Jalan Kerinchi, Bangsar South City, Kuala Lumpur 59200, Malaysia

**Keywords:** metabolomic profiling, Malaysian honey, New Zealander honey, metabolomic tools, multivariate analysis

## Abstract

This study aims to compare the metabolomic profiles of Malaysian and New Zealand honey while determining their anti-oncogenic activity for potential prophylactic functions. Metabolomics tools including multivariate analysis were applied on concatenated LC-HRMS and NMR datasets to afford an intensive chemical profile of honey samples and have a snapshot of the bioactive metabolites in the respective collections. Malaysian samples were found to have higher sugar and polyphenolic content, while New Zealand samples afforded higher concentration of low molecular weight (MW) lipids. However, New Zealand honey collected from the northern islands had higher concentration of acetylated saccharides, while those from the southern islands yielded higher low MW phenolic metabolites that were comparable to Malaysian honey. Mild anti-oncogenic compounds against breast cancer cell line ZR75 were putatively identified in Malaysian honey that included earlier described antioxidants such as gingerdiol, 2-hexylphenol-O-β-D-xylopyranoside, plastoquinone, tropine isovalerate, plumerinine, and 3,5-(12-phenyl-8-dodecenyl)resorcinol, along with several phenolic esters and lignans.

## 1. Introduction

Historically, honey has been used as food and cooking ingredient because of its sugars that serve as a source of energy [1]. Currently, honey has a wider role in therapeutic applications. It has been investigated for a variety of medicinal uses, including wound healing, cough, and skin allergies, as well as more advanced medicinal applications such as cancer [2]. Its anticancer activities were evidenced via in vitro and in vivo studies associated with breast, prostate, oral, renal, and cervical cancer [3,4,5,6]. A review published in 2009 summarised the anti-proliferative effect of honey and its polyphenol constituents, highlighting acacetin and kaempferol, which gave promising outcomes in the inhibition of human-lung cell lines, such as A549 and H460 [7].

There are several types of honey produced in Malaysia, famously known as Tualang, Gelam, Nanas and Acacia. These honeys were characterised based on the origin of their nectar. For example, Tualang honey was harvested and collected from the Tualang tree usually found in a Malaysian rainforest. Tualang honey has exhibited its effect as an anti-proliferative agent via the reduction of cell viability of oral squamous cell carcinoma (OSCC) and human osteosarcoma (HOS) cell lines due to apoptosis machinery [4]. Gelam honey, on the other hand, demonstrated activity as an anti-proliferative agent against the liver cancer line (HepG2), while it was found to be relatively nontoxic to a normal liver cell (WRL-68) [8]. Gelam honey potentially induced the anti-proliferative activity of cancer cells by triggering the apoptosis mechanism [8].

Several indigenous New Zealand honeys were characterised by their floral sources, such as *Leptospermum scoparium* (Manuka), *Kunzea ericoides* (Kanuka), *Trifolium* spp., (Clover), *Knightia excelsa* (Rewarewa), *Weinmannia racemosa* (Kamahi), *Ixerba brexioides* (Tawari), and *Thymus vulgaris* (thyme), as well as honey produced by the scale insects *Ultracoelostoma assimile* and *U. brittini* inhabiting the beech trees (Honeydew) *Nothofagus solandri* and *N. fusca* [9]. As reported in the literature, unifloral honey samples represent a respective plant species if the predominant plant is abundant in more than 45% of the geographical area [10]. The Manuka and Rewarewa honeys suppressed leukocyte infiltration via arachidonic acid-induced ear inflammation model in rats by a lower neutrophil level [11]. Meanwhile, the Kanuka honey showed high anti-inflammatory activity with the presence of a different level of methylglyoxal, whereby the neutrophil superoxide production remained unaffected. This demonstrated that high methylglyoxal levels do not suppress anti-inflammatory activity of the honeys. The Manuka and pasture honeys from New Zealand also showed a significant increase in cytokine (TNF-α, IL-1β and IL-6) release from isolated human peripheral blood monocytes that can promote wound healing [12]. Taken together, even though the therapeutic properties of honey have been well described in the literature, in contrast there is a lack of information available on Malaysian and New Zealand honeys for potential anticancer application. Therefore, the present study focused on a metabolite profiling study of Malaysia and New Zealand honeys based on the HR-LCMS, ^1^H NMR and their cytotoxic effect on A549, A2780, ZR75, and PANC-1, as well as their toxicity on normal cells, HFL-1.

## 2. Results

Metabolomics tools that include multivariate analysis were applied on concatenated LC-HRMS and NMR datasets to afford an intensive chemical profile of honey samples and to get a snapshot of the bioactive metabolites present in the respective collections according to their geographical origin. The obtained LC-HRMS data were processed with MZmine 2 and were dereplicated using an in-house Excel Macro to couple the MS data with the Dictionary of Natural Products database [13]. A supervised multivariate analysis was done by orthogonal partial least squares discriminant analysis (OPLS-DA) in SIMCA 15 to predict and pinpoint the plausible bioactive components against the breast cancer cell line ZR75.

### 2.1. Biological Activities of Honey Extracts

The 42 honey extracts from Malaysia and New Zealand demonstrated different bioactivities on various cancer and normal cell lines that included A549, A2780, ZR75, PANC-1 and HFL-1 at a concentration of 100 µg/mL (Table 1). Overall, the maximum cytotoxicity effect of all honey extracts was observed to reduce the viability of the normal human lung fibroblast cell line HFL-1 up to 71%. Therefore, the minimum cytotoxicity activity on cancer cell lines in this study was set to at least at 70% of control viability on cancer cell lines. Meanwhile, several of the Malaysian honey extracts, including CH, FH, GH, HH, JH, KH, LH, NH, RH, and SH showed a mild cytotoxicity effect on the breast cancer cell line ZR75, which afforded cell growth viability only by less than 50% (highlighted in red) while no bioactivity was observed on other cancer cell lines. New Zealand samples exhibited no cytotoxicity effect on ZR75 or any of the other cancer cell lines tested, which at the most afforded only 30% cell growth inhibition of ZR75 (Table 1).

### 2.2. Chemical Profiling of Malaysian and New Zealand Honey Based on ^1^H NMR Experiment

Proton signals at 3 to 6 ppm indicated the presence of a glucose moiety, as well as fructose within 3 and 4 ppm [14], the two major constituents of honey, albeit these chemical shifts can be generally assigned for sugars. An olefinic group was also predicted for proton signals between 2 to 5 ppm. In addition, the proton signals at 2.31 and 4.81 ppm, respectively, were assigned for methyl, and alkyl protons in methylglyoxal were observed in honey from New Zealand [15]. Taken together, the proton spectra of Malaysia and New Zealand (NZ) honey extracts were observed to have a similar chemical fingerprint (Supplementary Figures S1 and S2). The major compounds consisted of sugars, some of which were already converted to furfural and were subsequently oxidised to their acid form (Supplementary Figure S3) [16], as shown by the doublet signals [14] at 6.21 and 4.89 ppm, with coupling constants of 4.2 and 3.4 Hz, respectively. COSY NMR (Supplementary Figure S4) indicated the presence of the glucose moiety and 5-(hydroxymethyl)furan-2-carboxylic acid. A comparison of the proton NMR resonances of main constituents in the collected honey samples with those recorded in the literature [14,16,17,18] is summarized in Supplementary Table S1.

### 2.3. Chemical Diversity of Malaysian and New Zealand Honey Based on HR-LCMS Data

Base peak chromatograms of positive and negative ionization modes of honey extracts from Malaysia and New Zealand (NZ) are shown in Supplementary Figure S5a–d. After the removal of variable features from the solvent blank with intensity >1 × 10^5^, the Malaysian honey NH afforded the highest number of features, where 2743 and 2247 features were detected from the positive and negative ionization mode, respectively. Following dereplication, 27.8% (1383) of the combined features from both the positive and negative mode were putatively identified; whilst 72.2% (3593) remained unidentified, indicating that the sample may possibly contain novel compounds. For the New Zealand honey samples, the highest number of features was yielded by the honey sample BNZ, and 2720 features were detected in the positive mode and 2079 features were revealed in the negative mode. By dereplication, 28.6% (1369) of the combined features from both positive and negative modes were putatively identified, whilst 71.4% (3415) were unidentified. The total number of features identified from the HR-LCMS data from respective bioactive Malaysian and New Zealand honey extracts are presented in Supplementary Table S2.

Amongst the 42 honey samples, as mentioned above, NH and BNZ had the highest numbers of features and were chosen as a representative spectrum to identify the major ion peaks for the collected samples. Metabolites were putatively identified from the Dictionary of Natural Product (DNP) database for compounds previously isolated and described from plant sources indigenous to the origin of the samples collected (Table 2 and Supplementary Table S3). Along with various saccharides, sulphated furfural derivatives, such as those previously described from Chrysanthemum species [19,20], were amongst the major metabolites perceived in both Malaysian and NZ honey samples. The furfural derivatives detected in both positive and negative ionization modes, as shown in the mass spectra in Supplementary Figure S6a,b, were eluted within the retention time range between 0.69 and 2.27 min, when the percentage of organic mobile phase (acetonitrile) was at only 10%. Structures are presented in Supplementary Figure S7a,b. Moreover, major compounds from the Malaysian samples consisted of sugars and phenolics. On the other hand, in addition to sugars, the occurrence of glycosidic compounds was observed from NZ samples. But there were also several other intense ion peaks found exclusively in NH and BNZ honey samples, most of which were unidentified. Dereplicated natural products unique to NH and BNZ were vitelignin A [21] and 5,6-diglucopyranosylangelicin [22], which were first isolated from *Vitex negundo* and *Ficus ruficaulis* var. *antaoensis*, respectively. Vitelignin A has been described to exhibit cytotoxicity effects through the induction of apoptosis [6] and the ability to control metastasis [23].

### 2.4. Visualising Diversity between Malaysian and New Zealand Honey Using Heatmaps

**Figure 1:**
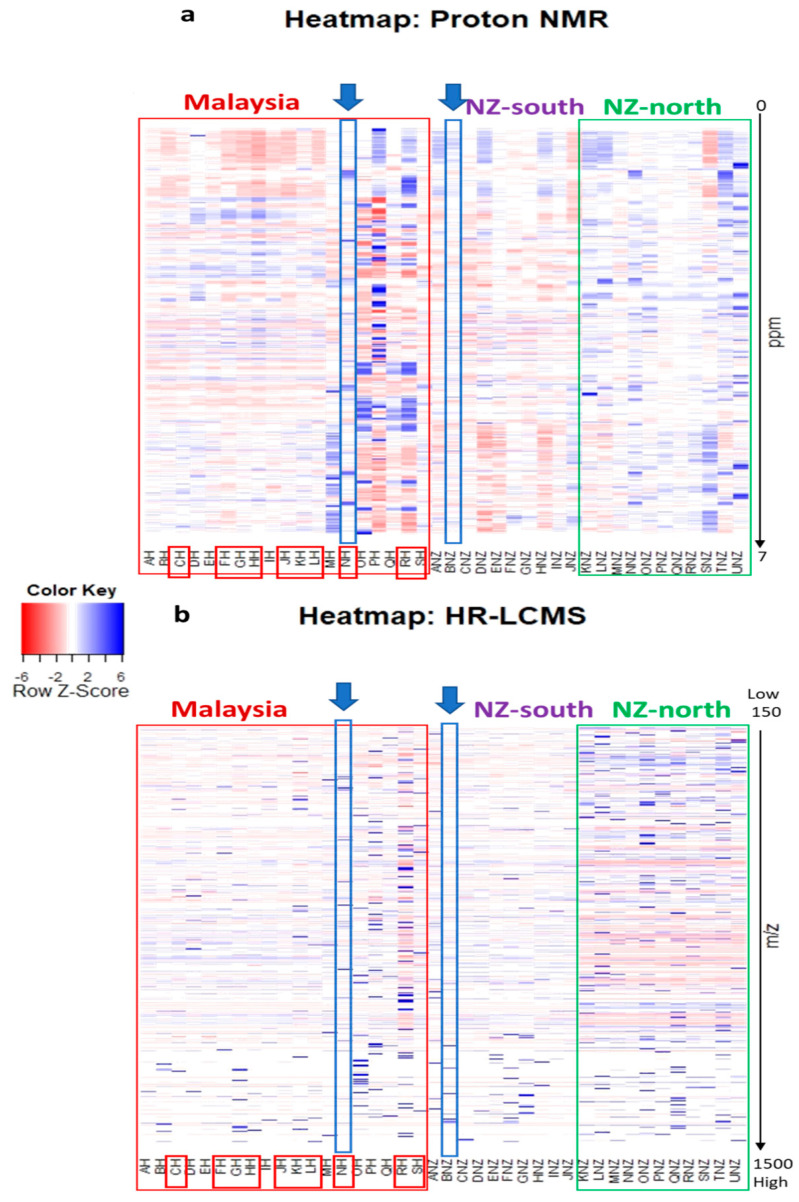
Heatmaps based on (**a**) ^1^H-NMR and (**b**) HR-LCMS data displaying distinct chemical profiles of 42 extracts, where the components highlighted in red box represent the samples from Malaysia. The components outside the red box are from New Zealand. Legend: **NZ-south** = South Island New Zealand honey, **NZ-north** = North Island New Zealand honey. Bioactive extracts were boxed in red. Enclosed in blue boxes under an arrow are samples NH and BNZ that have the greatest number of features identified by HR-LCMS. Bar intensities from red to blue, respectively, indicate the decrease and increase in concentration of individual metabolites in corresponding samples.

Heatmaps were further generated to better visualise and compare the chemical diversity of each individual samples as well as further establish the similarities and differences according to their geographical origin and perhaps their bioactivity as well. The heatmaps illustrated in Figure 1 were organised according to extracts showing differences in the chemical profiles at different intensity based on their ^1^H NMR and HR-LCMS datasets, respectively. The colour key from red to blue indicates increasing intensity of specific chemical constituents in the sample extracts.

Based on the proton NMR spectral data shown in Figure 1a, the Malaysian honey extracts disclosed an interesting chemical profile (boxed in red), especially those from MH, OH, PH and RH, affording a denser set of signals between 5 and 6 ppm. From the NZ honey samples, the North Island samples (NZ-north, boxed in green) yielded a more diverse chemical profile than the South Island samples (NZ-south). In addition, based on the mass spectral data displayed in Figure 1b, amongst the Malaysian samples (boxed in red) OH, PH, and RH were also observed to be the most diverse, where there is an increase in metabolites with MWs between 300 to 400 Da. However, only RH was deemed anti-oncogenic on the breast cancer cell line. For the NZ extracts, NZ-north samples were also found to have a more diverse chemical profile than NZ-south, which was a similar pattern, observed from the heatmap of the NMR spectral data. NZ-north (boxed in green) samples afforded low MW compounds ranging from 150 to 250 Da. However, samples NH and BNZ had the greatest number of features identified by HR-LCMS, where NH afforded higher cytotoxicity against ZR75.

### 2.5. Multivariate Analysis of Malaysian and New Zealand Honey Samples

#### 2.5.1. Principal Component (PCA) and Hierarchical Clustering Analysis (HCA)

**Figure 2:**
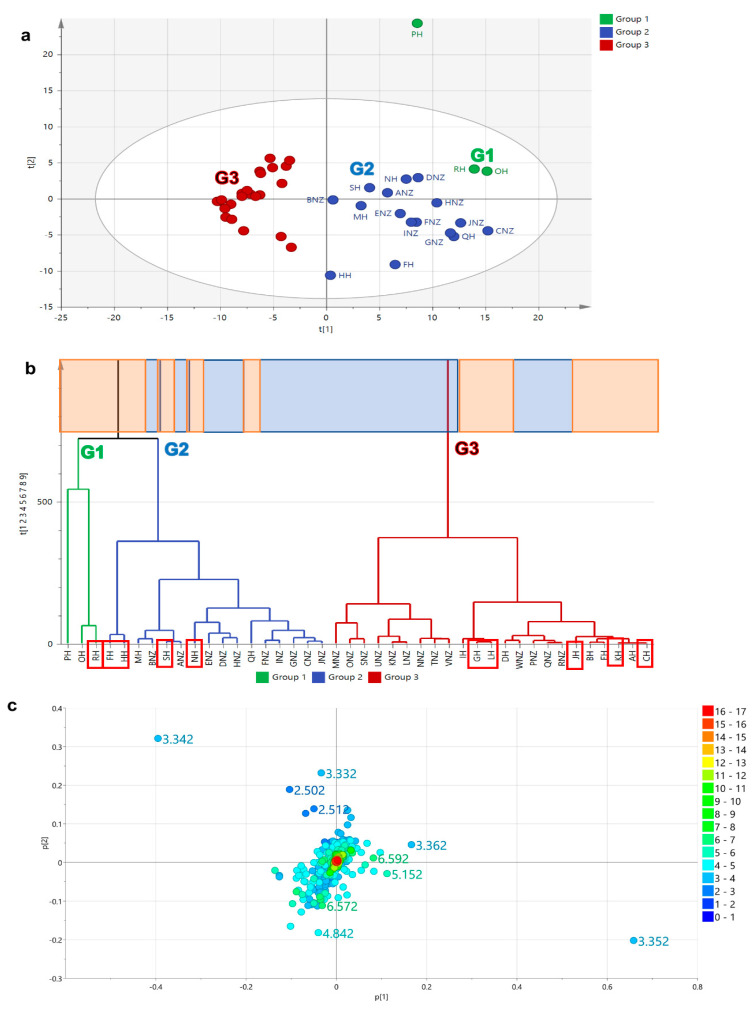
PCA model employing the NMR spectral dataset: (**a**) Scores, (**b**) HCA, and (**c**) loadings plots on honey extracts based on ^1^H NMR spectral dataset, which gave a relatively good model and prediction (R^2^ = 0.92 and Q^2^ = 0.68). Bioactive samples were boxed in red. Highlighted upper boxes on the HCA dendrogram (**b**) represents origin of honey samples where blue represents the New Zealand (NZ) samples while the Malaysian samples were highlighted in orange.

**Figure 3:**
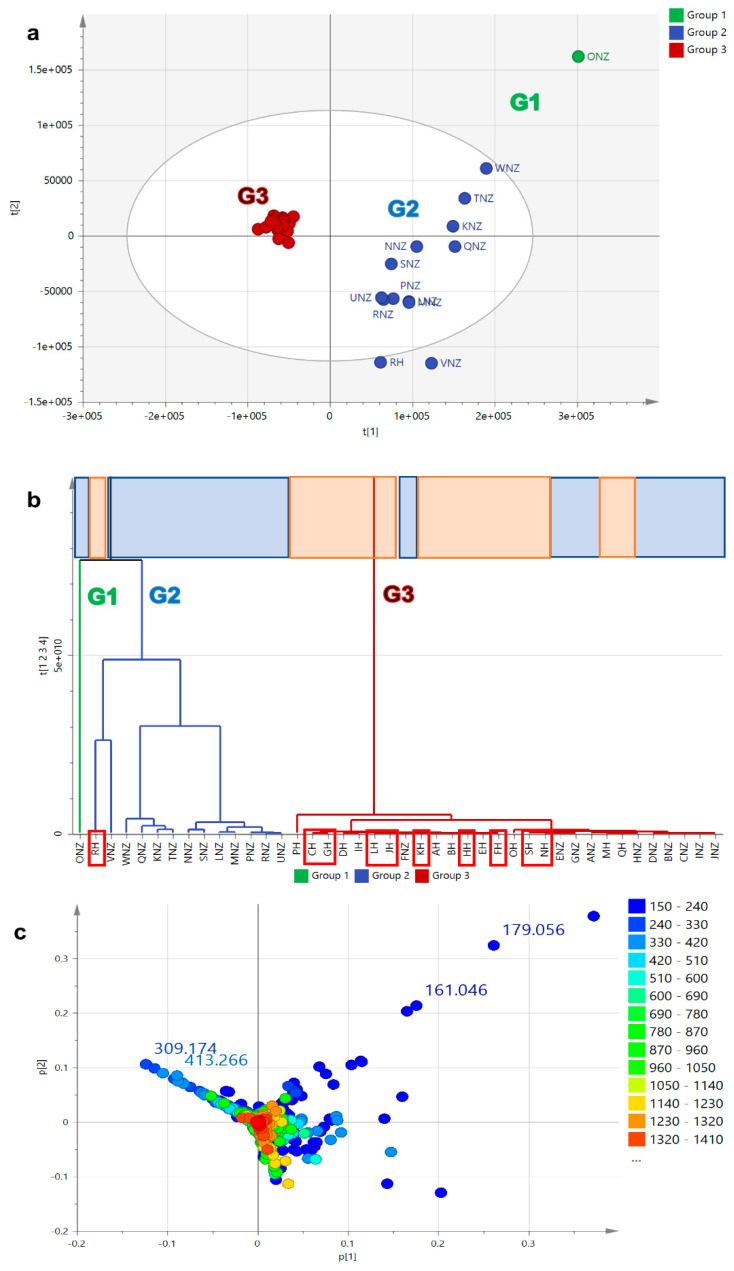
PCA model employing HRMS dataset. (**a**) Scores, (**b**) HCA, and (**c**) loadings plots and of honey extracts based on HR-LCMS (R^2^ = 0.60 and Q^2^ = 0.46). Bioactive samples were boxed in red. Highlighted upper boxes on HCA dendrogram (**b**) represents origin of honey samples where blue represents the New Zealand (NZ) samples while the Malaysian samples were highlighted in orange.

**Figure 4:**
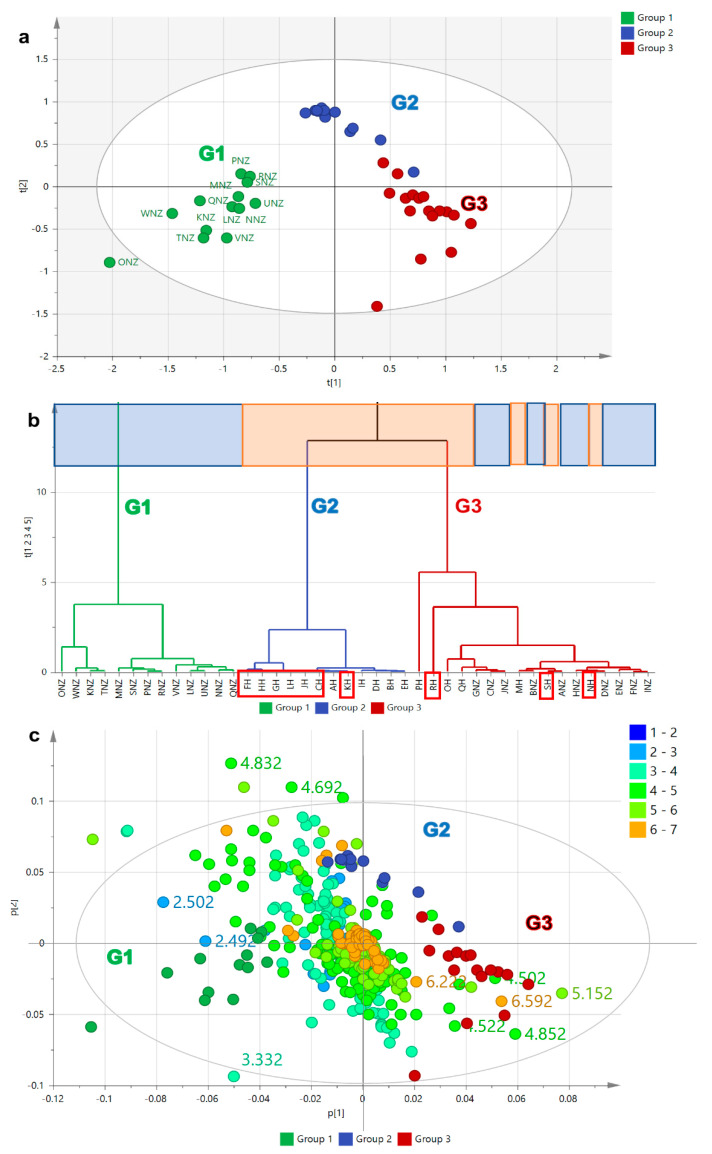
PCA model employing fused NMR and mass spectral datasets. (**a**) Scores and (**b**) HCA plots on honey extracts based on HR-LCMS and ^1^H NMR fusion which gave a fitness R^2^ value = 0.715 and predictive ability Q^2^ = 0.413. (**c**) PCA-biplot of the scores and extracted NMR loadings plot from the fused datasets. Bioactive samples were boxed in red. Highlighted upper boxes on HCA dendrogram (**b**) represents origin of honey samples where blue represents the New Zealand (NZ) samples while the Malaysian samples were highlighted in orange.

As mentioned above, the proton NMR spectra of both Malaysian and New Zealand (NZ) samples were observed to have similar major compounds. However, it was only amongst the Malaysian extracts that exhibited cytotoxic effects on ZR75 cell line, indicating a difference in chemical profiles between Malaysian and NZ honey samples. Therefore, an unsupervised multivariate analysis on the honey extracts using principal component (PCA) and hierarchical clustering analysis (HCA) was performed to verify the differences and similarities between the samples being analyzed following their natural distribution. Based on two datasets, multivariate analysis (Figure 2, Figure 3 and Figure 4) was done by employing three approaches. The first approach utilised only proton NMR spectral data (Figure 2), the second approach applied only the HR-LCMS data (Figure 3), and the third approach made use of the fused datasets of both HR-LCMS and ^1^H-NMR (Figure 4).

Clustering on the HCA was used to characterise and compare the chemical profiles of the samples in correlation to their bioassay results and geographical origin. PCA scores plot (Figure 2a) and HCA-dendrogram (Figure 2b) of the samples based on the proton NMR spectral data distributed the bioactive extracts in two main groups, G2 and G3, while the presence of an outlier from the Malaysian sample set (PH) could be observed in G1. The ^1^H NMR spectral dataset gave a relatively good model in terms of fitness and predictive ability. There was quite a variation observed between groups at 45% (R^2^X [1]), while within groups the variation score was only 18% (R^2^Xo [1]). The active Malaysian samples were distributed in G1 (RH), G2 (FH, HH, SH, NH), and G3 (GH, LH, JH, KH, CH). Both NZ and Malaysian samples were dispersed in both G2 and G3. Since NMR could only detect the major metabolites in the respective samples, despite the assigned groupings, it was not feasible to differentiate the samples according to their origin as well as predict the metabolites affecting the cytotoxicity of the samples on ZR75. The loadings plot (Figure 2c) also did not clearly differentiate the resonances between the groups.

PCA-scores plot (Figure 3a) and HCA-dendrogram (Figure 3b) of the HR-LCMS dataset indicated a similar clustering pattern, which also resulted to two big main groups, G2 and G3 along with an outlying group (G1) consisting of a lone sample, which for this model was ONZ from New Zealand. The mass spectral dataset gave a relatively weaker model in terms of fitness and predictive ability but was not over-fitted with an R^2^/Q^2^ difference of 0.14, which is ≤0.3. Variation observed between groups increased to 49% (R^2^X [1]), while within groups it decreased to 10% (R^2^Xo [1]). The bioactive samples were mostly distributed in G3 except for RH that was found in G2. These results were compatible with those achieved from the heatmap analysis of the samples that visually explained the outlying character of the sample RH in both NMR and mass spectral datasets. However, there was a better separation between the Malaysian and NZ samples when employing the mass spectral dataset. G2 clustered 56% of the NZ samples (highlighted in blue), while G3 had 94% of the Malaysian samples (highlighted in orange), which were more tightly clustered when compared to the NMR datasets. Additionally, it can also be observed that the Malaysian samples in G3 as shown in Figure 3b had most of the anti-oncogenic active samples (enclosed in red boxes). It could be said the mass spectral data indicated that the presence of low-concentration metabolites was responsible in differentiating between Malaysian and NZ samples. As revealed by the PCA-loadings plot (Figure 3c), the discriminating metabolites for NZ samples had low MW metabolites ranging from 150 to 250, while the Malaysian samples had unique metabolites with higher MWs from 300 to 420. The discriminating metabolites for NZ and Malaysian samples were listed on Table 2. Malaysian discriminating metabolites were from semi- to non-polar compounds with retention time ranging from 15 to 40 min, while those from NZ were very polar metabolites eluting before 5 min.

The clustering observed in the PCA scores (Figure 4a) and HCA dendrogram (Figure 4b) for the concatenated MS and NMR datasets was comparable to employing the mass spectral dataset on its own. The fitness of the model was improved, but the predictive ability remained relatively the same while still considered not to be overfitted with a R^2^/Q^2^ difference of 0.30. The samples were grouped into three clusters, G1, G2, and G3, with an approximately equal number of observations and disappearance of an outlying sample. G2 clustered 56% of the NZ samples (highlighted in blue) while G3 had 84% of the Malaysian samples (highlighted in orange), which decreased by 10% when compared to employing just the mass spectral dataset. G3 clustered 40% of the entire number of observations. R^2^X [1] and R^2^Xo [1] variations were determined at 34% and 17%, respectively, indicating less variation between groups and increased variation within groups when compared to the two latter approaches. The mass spectral features followed an identical trend as the unfused model, but the density of the mass spectral dataset concealed the positions of the NMR spectral features with its lower number of datapoints. The NMR datapoints were then extracted on the biplot (Figure 4c) to be able to visualise their positions on the respective quadrants. Extraction of the NMR features from the concatenated datasets (Figure 4c) revealed a higher density of upfield resonances between 1 to 3 ppm for the NZ samples (G1), which indicated the presence of low MW lipids. Alternatively, the Malaysian samples (G2) seem to be dominated by downfield signals between 3 to 5 ppm indicative for sugar compounds, while the G3 samples contain more signals from the 5 to 7 ppm region, which could belong to phenolic or other aromatic compounds. From the fused datasets, it was possible to define the NMR resonances that exclusively belong to the respective geographical sources.

#### 2.5.2. OPLS-DA Based on Geographical Area of Collection

**Figure 5:**
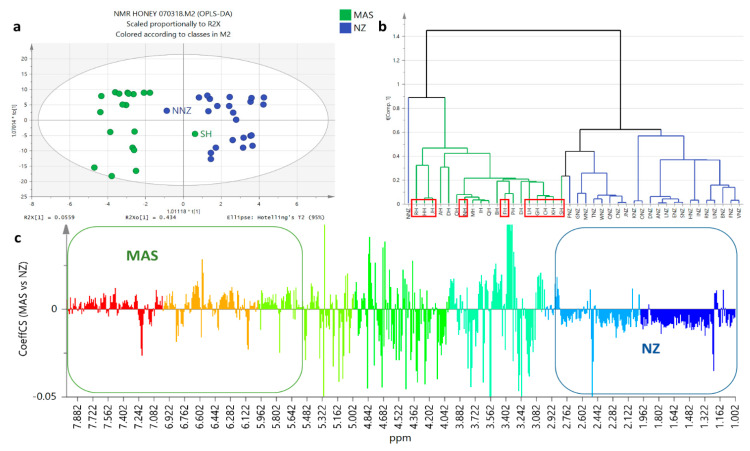
OPLS model employing the NMR spectral dataset. Bioactive extracts were boxed in red. (**a**) OPLS-DA scores plot (R^2^ = 0.84 and Q^2^ = 0.59). Permutation test generated a Q^2^Y = −0.682. (**b**) HCA by single linkage, and (**c**) coefficient plot of ^1^H NMR spectral data of honey extracts. Legend: MAS (Malaysian samples), NZ (New Zealand samples).

**Figure 6:**
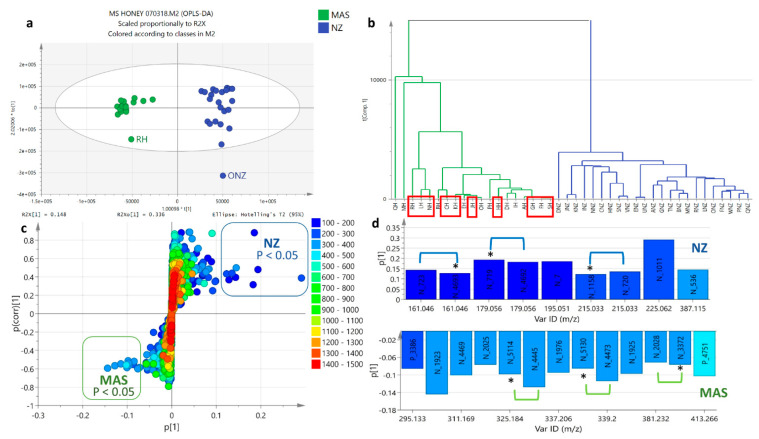
OPLS model employing the mass spectral dataset. Bioactive extracts were boxed in red. (**a**) OPLS-DA scores plot (R^2^ = 0.91 and Q^2^ = 0.64). Permutation test generated a Q^2^Y = −0.355. (**b**) HCA by single linkage, (**c**) S-loadings plot of mass spectral data of honey extracts, and (**d**) Coefficient bar graph showing the discriminating metabolites with *p* < 0.05 as boxed on the S-plot. * Due to identical RT and *m/z* values with another variable feature, only the one with a higher *p* value was included in Table 2. Legend: MAS (Malaysian samples), NZ (New Zealand samples).

**Figure 7:**
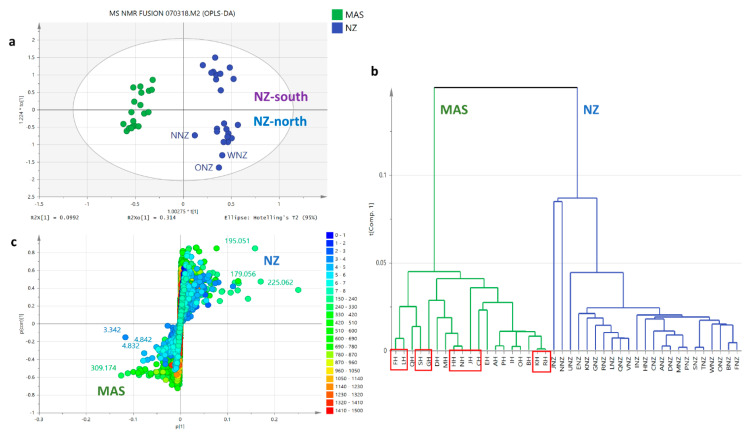
OPLS model employing fused NMR and mass spectral datasets. Bioactive extracts were boxed in red. (**a**) OPLS-DA scores plot (R^2^ = 0.997 and Q^2^ = 0.974). Permutation test generated a Q^2^Y = −0.473, (**b**) HCA by single linkage, and (**c**) S-loadings plot of ^1^H NMR and mass spectral data of honey extracts. (R^2^ = 0.96 and Q^2^ = 0.74). Legend: MAS (Malaysian samples), NZ (New Zealand samples, color codes on S-plots are indicated by their chemical shifts in ppm values from 0 to 8 ppm and ion peaks at *m/z* from 150 to 1500 Da.

**Figure 8:**
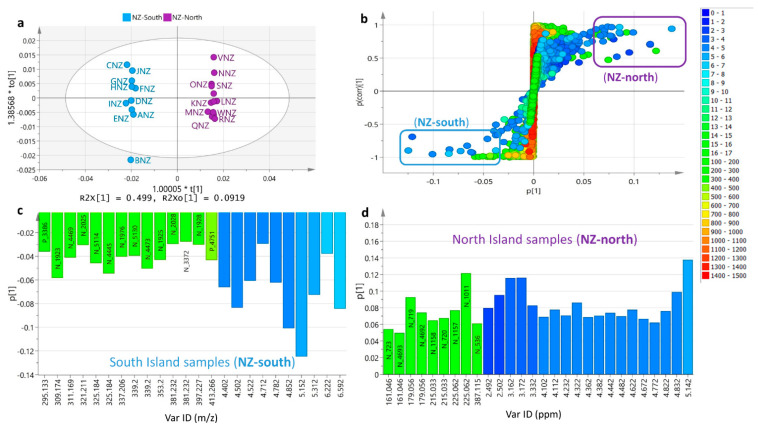
Comparison between honey samples from North and South Islands. (**a**) OPLS-DA scores plot, (**b**) S-loadings plot of mass spectral data of honey extracts, and coefficient bar graphs showing the discriminating metabolites for the North (**c**) and South (**d**) Island samples as exhibited at the “end points” of the S-plot. Permutation test generated a Q^2^Y = −0.552. Color codes on S-plots are indicated by their chemical shifts in ppm values from 0 to 17 ppm and ion peaks at *m/z* from 150 to 1500 Da.

An OPLS-DA analysis (shown below in Figure 5, Figure 6, Figure 7 and Figure 8) was performed on Malaysian and NZ samples to confirm the discriminating metabolites found in PCA as based on their geographical origins. Based on the proton NMR spectral data, the OPLS-DA scores plot (Figure 5a) affords a good model with a R^2^/Q^2^ difference of 0.25, which is considered not to be overfitted. Both R^2^X [1] and R^2^Xo [1] variations were determined at 6% and 43%, respectively, indicating a very small variation between the Malaysian and NZ samples, while there was an increased in variation within the respective groups. The small variation between Malaysian and NZ samples could be deduced by two extracts, SH and NNZ, belonging to Malaysian and NZ, respectively. SH and NNZ crossed over the quadrants of the respective groups as also shown on the HCA dendrogram (Figure 5b). The OPLS-DA coefficient plot (Figure 5c) gives a clearer distribution of the resonances, which is still comparable to the results obtained with the extracted NMR features from the PCA of the fused datasets (Figure 4c). Figure 5c demonstrates the occurrence of upfield resonances between 1 to 3 ppm for the presence of lipids in NZ samples. Alternatively, the Malaysian samples afforded an increase in number of signals in the downfield region between the 5 and 7 ppm region, which could be evidence for the occurrence of polyphenolic compounds in the samples. The presence of a hydroxyl substituent in a phenyl ring would shield protons to be resonating upfield from 7.25 ppm. Furthermore, between 3 to 5 ppm, the so-called sugar region, NZ samples seem to contain more intense signals than the Malaysian samples, indicating that the NZ honey extracts had higher sugar content than Malaysian honey.

Analysis of the mass spectral datasets by OPLS-DA (Figure 6a,b) indicated a comparable clustering pattern as that obtained by PCA, which gave the same outliers, RH and ONZ, from their respective groups. The loadings plot showed dominant occurrences of compounds with MWs between 280 and 420 g/mol for Malaysian samples observed on the lower left quadrant of the S-plot (Figure 6c) and listed in Table 2a. The discriminating metabolites between the two geographical classes were considered significant at *p* < 0.05. The structures of dereplicated compounds are shown on Supplementary Figure S7A,B. The higher MW compounds could be predicted to be phenolic compounds as endorsed by their NMR spectral data as well with resonances between 5 to 6 ppm (Figure 5c). The right upper quadrant had higher incidences of smaller MW compounds ranging from 150 to 280 g/mol, which may indicate low MW lipids and sugars (mono and disaccharides) in NZ honey that would be compatible with the upfield resonances in the NMR results. The discriminating metabolites generated by PCA and OPLS-DA were comparable but not identical to each other. The discriminating metabolites were listed according to their *p* values on Table 2 and plotted on a bar graph in Figure 6d. The list specified additional metabolites that differentiated Malaysian from NZ samples. The list also showed that there was a higher incidence of metabolites with even-numbered MW and ionizing in the negative mode, which further indicated the presence of low MW fatty acids and sugars [24] for the NZ samples as well as phenolics in Malaysian samples.

On top of that, the best clustering was observed after fusion of the MS and NMR spectral datasets as shown in Figure 7a,b. In comparison to the latter two approaches, the model was the best fit and gave the best predictability. At least three clustered groups were observed where all the Malaysian extracts were clustered in one group, whilst there were two independent groups detected for NZ extracts as shown on the right quadrant of the scores plot (Figure 7a). However, the occurrence of two subgroups for the NZ samples was not properly manifested on the HCA (Figure 7b) where samples between the two subgroups cross-linked each other. The occurrence of two subgroups for the NZ samples gave a high variation within groups at 31%, while between the two specified sample groups, Malaysian and NZ, the variation was only 9.9%. With no outliers, the percentage variation for the fused dataset was comparable to the OPLS-DA model for the mass spectral data. As shown on the loadings S-plot (Figure 7c), Malaysian honey has a higher sugar and polyphenolic content represented by the higher density of lighter blue dots, while the greater density of yellow-green to orange spots indicated higher MW compounds. Alternatively, New Zealand honey has higher low MW lipid content represented by the higher density of darker blue dots, while the higher density of lighter green spots indicated lower MW compounds. Interestingly, in the fused datasets, both groups from NZ extracts were further clearly subclustered based on the two main islands of New Zealand, where the upper right quadrant enclosed the South Island samples (NZ-south), particularly Awatere Valley, Wairau Valley, Linkwater and Moutere; in the lower right quadrant (NZ-north), samples from the north island of New Zealand clustered together. However, the specific information about the geographical area was not provided by the supplier and therefore the result only relies on the trend observed from the OPLS-DA analysis. Furthermore, the OPLS-DA scores plot (Figure 7a and Figure 8a) along with its corresponding S-plots (Figure 7c and Figure 8b) afforded better clustering, indicating differences in their chemical fingerprint.

To differentiate the samples collected from the South (NZ-south) and North (NZ-north) Islands in New Zealand, an OPLS-DA was performed on the fused NMR and mass spectral dataset of the NZ samples (Figure 8a). A very good and valid model was attained, while the permutation test afforded a Q2Y of −0.552. The occurrence of two distinct groups for the NZ samples increased the variation between groups at 50%, while within groups, the variation was down to 9% indicating a homogenous set of samples for the respective South Island (NZ-south) and North Island samples (NZ-north). However, BNZ from the NZ-south group came as an outlier, which afforded the highest number of features as earlier mentioned in this study. The S-plot (Figure 8b) revealed the features that differentiated NZ-south from NZ-north. The discriminating features, with *p* values < 0.05, were extracted and shown in coefficient bar graphs for NZ-south and NZ-north (Figure 8c,d), respectively. The samples collected from the South Island (NZ-south) gave higher MW metabolites (*m*/*z* at 250 to 400 Da) and indicated the presence of phenolic compounds with NMR resonances between 6 to 7 ppm. The North Island samples (NZ-north) were more distinct and were discriminated by low MW compounds (*m*/*z* at 150 to 240 Da [M−H]^−^) and NMR chemical shifts at 2 to 3 ppm, which could indicate the presence of acetylated sugar or furfural molecules.

The discriminating ion peaks for the South Island (NZ-south) samples matched those features that differentiated Malaysian from NZ honey. Interestingly, all the pinpointed metabolites that were defined as unique for the Malaysian samples were also responsible for demarcating the NZ-south samples. As illustrated on the coefficient bar graph for the NZ-south samples (Figure 8c), these metabolites were semipolar (RT: 15–40 min) and phenolic (^1^H-NMR shifts at 4–7 ppm) with MWs between 250 and 400 Da. The ion peak at *m*/*z* [M−H]^−^ 309.17 (N_1923 at 16.15 min) was putatively identified both as phenolics (gingerdiol and 2-hexylphenol-O-β-D-xylopyranoside) and sesquiterpenoids (blumeaene L and 9-acetyl-6,7-dihydroxy-3(15)-caryophyllen-8-one). The ^1^H-NMR resonances between 4 and 7 ppm could imply that dereplication hits towards gingerdiol or 2-hexylphenol-O-β-D-xylopyranoside would be more compatible than those of the sesquiterpenoids. Hence, this also explained the overlapping of the Malaysian and NZ samples under G3 in the PCA scores (Figure 4a) and HCA (Figure 4b) plots of the fused datasets. The NZ-south chemical profile was quite like those of the Malaysian samples, which however, were still not identical as projected by their difference in biological activity profile. The only discriminating feature that was found solely amongst the NZ-south samples and not detected in Malaysian honey was the ion peak at *m*/*z* [M−H]^−^ 397.227 (N_1928 at 22.19 min), which was not identified under this study. In addition, features with Mzmine IDs of N_3372, N_5114, and N_5130 with ion peaks at *m*/*z* 381.23, 325.184, and 339.2, respectively, which were like those found in the Malaysian samples, showed retention times with a difference of only 0.2 to 1.4 min.

Alternatively, the North Island (NZ-north) samples afforded the identical set of discriminating features for the NZ samples in its entirety when differentiated from Malaysian honey samples. Similarly, as demonstrated on the coefficient bar graph for the NZ-north samples (Figure 8d), the discriminating features were polar (RT: 1–5 min) and acetylated saccharides (^1^H-NMR shifts at 2.5–5 ppm) with lower MWs between 150 and 300 Da. OPLS-DA classification based on geographical area of collection gave better interpretation on the type of compounds found in Malaysian and NZ honey as well as the separation between the samples collected from the North and South Islands of New Zealand.

### 2.6. Dereplication of Bioactive Metabolites

**Figure 9:**
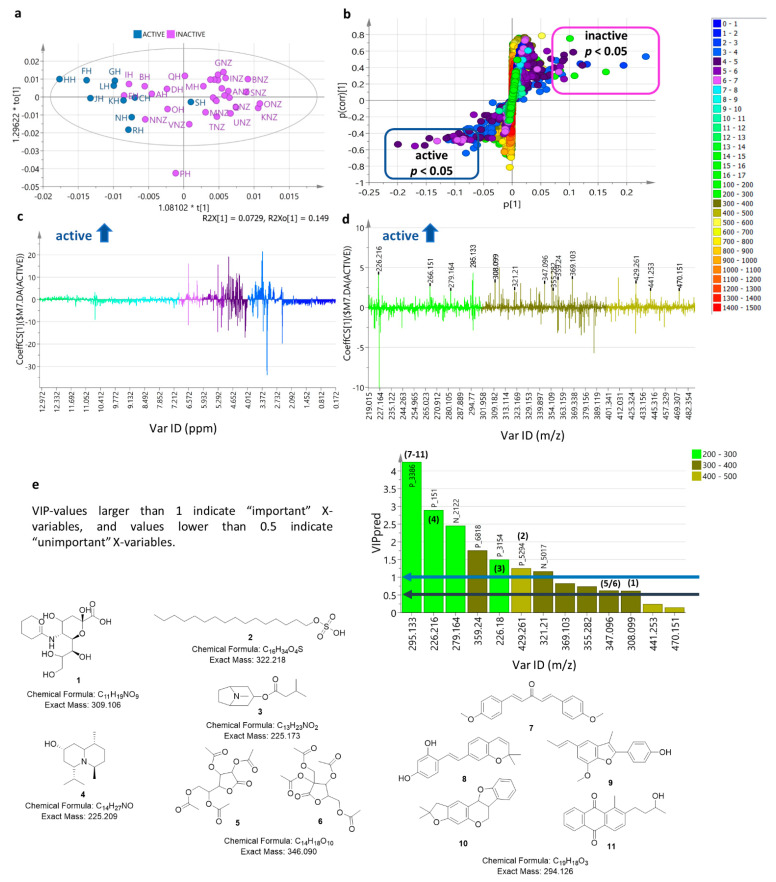
OPLS-DA model for bioactivity of honey samples against ZR75. (**a**) Scores plot of active versus inactive samples. (**b**) S-loadings plot pinpointing the putative bioactive metabolites in active extracts. (**c**) Coefficient plot indicating the ppm chemical shifts for functional groups of bioactive metabolites as shown on the positive phase of the Y axis. (**d**) Coefficient plot showing discriminating ion peaks *m*/*z* for bioactive samples, where labelled peaks have *p* < 0.05 as listed under Table 2c. (**e**) VIP plots and dereplicated structures of ion peaks for the bioactive variable features. Blue arrow indicates threshold for metabolites with VIP values > 1.0 while black arrow designates compounds with VIP values < 0.5. Color codes on S-plots are indicated by their chemical shifts in ppm values from 0 to 17 ppm and ion peaks at *m*/*z* from 150 to 1500 Da.

Furthermore, the type of metabolites involved in the bioactive fractions against the breast cancer cell line ZR75 were determined by OPLS-DA (Figure 9), and by using an S-loadings plot (Figure 9b) it was then plausible to pinpoint the bioactive metabolites that could be putatively responsible for the bioactivity.

The active and inactive extracts were compared using the OPLS-DA scores plot (Figure 9a). R^2^ and Q^2^ showed poor fitness and prediction ability at two components. The permutation test afforded a Q^2^Y value of −0.257, which still supported the validity of the model but could be overfitted due to the large difference between R^2^ and Q^2^ that was >3. In addition, both R^2^X [1] and R^2^Xo [1] were determined at 0.073 and 0.149, respectively. This also indicated that the variation score between active and inactive was at only 7.3%, which was smaller than the variation score within group at 15%. This could be explained by the occurrence of the inactive samples crossing over the left quadrants for the active samples. On the other side, FH amongst the active sample was also positioned on the inactive right quadrants. Meanwhile, the larger variation score within the group was not only because of the overlapping positions of the active and inactive samples but also due to the occurrence of an outlier sample (PH) as shown in the scores plot (Figure 9a). From the scores plot alone, it was difficult to define the chemical differences between the samples and correlate them to the bioactivity of a few of the Malaysian samples. The S-loadings plot (Figure 9b) was then used to identify the putative bioactive metabolites that could be responsible for the bioactivity of the samples. Comparison of the active and inactive end points of the S-plot indicated a higher density of resonances between 4 and 7 ppm with MWs from 300 to 400 Da for the cytotoxic metabolites against ZR75. These differences were better visualised by employing coefficient plots (Figure 9c,d). The dereplication database of the predicted bioactive metabolites was presented in Table 2c.

The determined bioactive metabolites ranged from polar to nonpolar compounds with MWs between 200 and 500 Da. To validate the significance of these compounds, their *p* values must be less than 0.05 and VIP (Variable Importance in Projection) values must be greater than 1 (Figure 9e). In view of both the required *p* and VIP values cut-off, the pinpointed bioactive metabolites were achieved for ion peaks at *m*/*z* [M+H]^+^ 226.180, 226.216, 295.133, 359.240, 429.261, and [M−H]^−^ 279.164, and 321.210, three of which remained unknown (*m*/*z* 279.164, 359.24, and 321.21), while ion peaks at *m*/*z* [M−H] ^−^ 441.253 and 470.151 were designated to be “unimportant” variable features. Structures of the dereplicated compound were listed in Table 2c, and their structures are shown in Figure 9e.

Remarkably, except for P_3386, an ion peak at *m*/*z* [M+H]^+^ 295.133 (RT at 19.25 min), none of the discriminating metabolites for Malaysian honey were found to be amongst the putatively identified bioactive metabolites against ZR75. P_3386 was dereplicated with more plausible hits, but only the metabolites that have been described from plants distributed from peninsular Malaysia were considered (Table 2). P_3386 was found to be the discriminating feature for Malaysian honey with a *p* value of 3.11 × 10^−4^, but also differentiated the South Island (NZ-south) samples at *p* = 8.15 × 10^−15^. In terms of bioactivity, a *p* value of 3.75 × 10^−2^, P_3386 afforded the highest VIP value of 4.5, implying the most important X-variable in terms of bioactivity of the extracts. However, the presence of P_3386 in NZ-south honey did not make these New Zealand samples biologically active. This could imply that the occurrence of unique metabolites in the other anti-oncogenic samples could have played an essential role in the bioactivity of P_3386.

## 3. Discussion

Honey predominantly consists of sugars and other organic compounds. In this study, in addition to galactose and glucose, furfural and amino derivatives were identified as major compounds in the honey extracts based on their high-resolution mass spectral data. Furfural and sugar derivatives, as major compounds, were dereplicated with the DNP database from the honey samples through combined assessment of their proton NMR and HR-LCMS spectral data. Honey samples from both countries share one major compound, which is 1-(2,3-dihydro-2-furyl)-4-(thien-2-yl)but-1-en-3-yne.

### 3.1. Geographical Differences of Collected Honey Samples

From this study, honey chemical profile differences were observed according to their geographical differences. Peninsular Malaysian vegetation is a tropical rainforest dominated by dipterocarps from lowland to low hills between an altitude of 762 and 1200 m above sea level (https://www.malaysia.gov.my/portal/content/143, accessed on 6 January 2022). The New Zealand vegetation, with its abundant rainfall, is very different from that of Malaysia, where there is a general distribution of manuka, conifers, lycopods, tree ferns and other ferns found in its shady forests. However, variations in habitats are evident within short distances as the sea level and snow line changes, and there is an observable difference between the North and the South Islands. The North Island vegetation grows well in warm, humid areas on a variety of substrates, from volcanic soils to sandstone, but gives way to broadleaf forests in the more fertile valleys [25]. Kauri forests (*Agathis australis*) used to cover most of the North Island, but today only isolated stands remain. Subtropical rainforests are found on the North Island [26].

On the other hand, the lowlands and hills of the South Island of New Zealand are covered by a dense temperate rainforest usually dominated by podocarps until 400 m and silver beech at 800 to 1000 m thriving in colder subalpine and alpine weather [26]. The temperate rainforests of the South Island support a rich flora of over 220 species of higher forest plant that have been recorded on the southern coast. The wettest area of New Zealand is on the west coast of the South Island (https://www.fergusmurraysculpture.com/new-zealand/temperate-rain-forests/, accessed on 6 January 2022).

Multivariate analysis of fused or concatenated datasets by PCA and OPLS improved the recognition of the differences in chemical profiles between the honey samples grouped according to their geographical origin and bioactivity, which appeared to be more similar as biomarker metabolites were imperceptible by common major metabolites. Subjecting the fused datasets for multivariate analysis improved the fit, predictive ability, and variation scores of the models that resulted from the decreased dispersal of the samples within groups. In parallel, the differences between subgroups were enhanced as those observed for the North and South Island samples of New Zealand, which were not properly defined and indistinct when analysing separated spectral datasets. From the results of the PCA of the fused datasets, there was an overlapping of the NZ-south samples (ANZ to JNZ) with those of the Malaysian samples, three of which were active (NH, RH, and SH), collected from Malacca and Sarawak. Except for DH, all the other inactive Malacca samples also did cluster with NZ-south samples under G3. The rest of the inactive samples collected outside of Malacca were found in G2 along with the other active samples gathered from other regions. This indicated the occurrence of a subset of marker metabolites that are not necessarily anti-oncogenic but were predicted to be commonly found in honey samples from Malacca and New Zealand’s South Island. It is also noteworthy mentioning that unlike the North Island Manuka blend samples, South Island honey samples were in combination with other plant sources such as clover, honey dew, and borage. These marker metabolites were predicted as semipolar phenolic compounds with MWs between 250 and 400 Da, putatively identified as gingerdiol and 2-hexylphenol-O-β-D-xylopyranoside described from *Zingiber* species and *Leucas aspera,* respectively or sesquiterpenoid 9-acetyl-6,7-dihydroxy-3(15)-caryophyllen-8-one reported from *Buddleja davidii*. Antimicrobial phenolic trihydroxyflavanones [27] and caryophyllene [28] derivatives have also been described from *Leptospermum* species. Alternatively, *Buddleja davidii* has been categorised as an invasive weed currently causing problems in New Zealand. Compared to the North Island rainforests, the South Island has been amply modified by men and animals [26], i.e., agriculture that includes pastoral farming. Furthermore, the introduction of more robust but invasive angiosperms replacing dying old podocarps is resulting to regeneration failure. Podocarp saplings are light-demanding and will have difficulty regenerating in comparison to, e.g., a ginger plant that would thrive more easily on a shady forest floor.

### 3.2. Metabolites from Malaysian Honey

Of the 19 honey samples from Malaysia (MAS) analyzed in this study, 10 of them exhibited bioactivity on ZR75. The anti-oncogenic honey samples collected from different areas in Malaysia ascribed to be unifloral sources including *Acacia* sp. (CH), *Koompassia excels* locally known as Tualang (FH), *Momordica charantia* or bitter gourd (GH), *Melaleuca* sp. or paperbark (HH), *Asystasia gangetica* also known as Chinese violet (JH), *Acacia mangium* known as black wattle (LH, NH and SH), and from multifloral sources (KH and RH). Different geographical areas can provide both uni- and multifloral forage opportunity to a variety of bee species such as *Apis trigona* sp. (CH, KH and RH), *Apis dorsata* sp. (FH) and *Apis mellifera* sp. (GH, HH, JH, LH, NH and SH).

From the dereplication study, we can trace the potential plant source of the collected nectars. However, while some predicted features of bioactive metabolites remain unidentified, putatively identified metabolites did not match those of the recorded unifloral sources. Albeit 80% of the active honey samples were observed to be collected from unifloral sources, those collected from agricultural estates such as AH, BH and MH were rendered inactive—those for which the nectar sources were from the Pará rubber tree, cinnamon, and pineapple, respectively. Yet the question remains how selective or specific were the bees collecting the nectar from these respective unifloral samples.

Amongst the putatively identified metabolites unique to Malaysian honey samples included earlier reported antioxidants such as gingerdiol [29] and 2-hexylphenol-O-β-D-xylopyranoside [30] isolated from *Zingiber officinale* and *Leucas aspera*, respectively; cetylsulfate, a known natural product from *Cocos nucifera*; plastoquinone congener previously described from *Spinacia oleracea* [31]; and 5-(12-phenyl-8-dodecenyl)resorcinol reported from *Knema laurina*, also known as black wild nutmeg [32] that is widely distributed in peninsular Malaysia. *K. laurina* extracts were reported to exhibit highly anti-inflammatory and neuroprotective effects [33]. Except for P_3386, none of these discriminating features were designated as bioactive variables. In addition, an ion peak at *m/z* 321.210 putatively identified as cetylsulfate was also assigned as a discriminating metabolite for a bioactive feature. However, the latter ion peak had a Mzmine ID of N_2025 eluting at 29.19 min that could be a structural isomer of the bioactive metabolite, N_5017, with an RT of 37.32 min, where there is a plausible change in position of the sulfate unit in the alkyl chain as in dioctyl sulfate.

Some of the ion peaks designated to be the biologically active metabolites were dereplicated as cetylsulfate (**2**) from *Cocos nucifera*, tropine isovalerate (**3**) from a tumor inhibiting extract of the mangrove plant *Bruguiera sexangular* [34], and the lupine alkaloid; plumerinine (**4**) from an antioxidant extract of *Plumeria rubra* [35,36]. The sodium salt of cetylsulfate (**2**) has been described to be active in several yeast strains with defined genetic alterations in the NCI Yeast Anticancer Drug Screen [37]. *O*-sialic acid (**1**) and tetra-acyl lactones (**5** and **6**) gave low VIP scores at <1.0. Although they were amongst the selected bioactive variables with significant *p* values of 1.11 × 10^−4^ and 3.54 × 10^−2^, respectively. P_3386 found at *m*/*z* 295.133 (C_19_H_18_O_3_), eluting at 19.25 min, was statistically classified as the most “important” X-variable amongst the active features and was dereplicated with several hits that mostly possess antioxidant polyphenolic ester or lignan structures that could contribute to the anti-oncogenic activity of the honey samples [38], such as dianisylidene acetone (**7**) from *Curcuma longa*, 5-dehydroxyartocarbene (**8**) from *Artocarpus incises* (breadfruit), eupomatenoid 13 (**9**) from *Caryodaphnopsis tonkinensis*, and methyloroxylopterocarpan (**10**) from *Oroxylum indicum* except for sterequinone H (**11**), which is an anthraquinone from *Stereospermum personatum*. All these plant sources are widely distributed in peninsular Malaysia. These putatively identified hit compounds would need to be chromatographically isolated to further prove their structures by NMR and validate the bioactivity, which was not feasible in this study due to the very low yield.

Tualang honey was evident to give strong antioxidant activity from the ferric-reducing ability of a plasma (FRAP) assay [39]. Several studies demonstrated the positive modulation effects of Tualang honey from Malaysia on the breast cancer cell line MCF-7 and DMBA/MNU-induced breast carcinogenesis in Sprague Dawley rats [40,41,42,43]. However, the benefit of honey as an anticancer modulating agent has increased arguments, since honey has high levels of carbohydrates in its concentrated form. This could yield high levels of calories that could initiate cancer formation, especially in breast cancer [44]. Nevertheless, Tualang honey consumption in DMBA-induced breast cancer in rats was earlier described in the literature not to have a major influence in the test animal’s body weight [43]. To date, there has been no phytochemical work on the plant source *Koompassia excels.*

### 3.3. Metabolites from New Zealand Honey

The New Zealand honey samples, in this study, were predominantly collected by the *Apis mellifera* bees. One of the sources of its nectar was the Manuka tree along with other plant sources that include clover (ANZ, BNZ, and JNZ), honey dew (ANZ and JNZ) and borage (BNZ), as well as a mixture of other plants within the foraging area of the bees (MNZ, ONZ, QNZ, and WNZ). Maximum efficiency of Manuka honey at 0.6% concentration has been demonstrated to inhibit at least ≤90% of cell viability in breast cancer cell line (MCF-7) after 72 h treatment [45], while, a co-treatment using Manuka honey and paclitaxel provided better control of tumor growth and improved host survival in a melanoma mouse model [45]. Meanwhile, oral administration of Manuka honey at 1.0 g/kg body weight/day continuously for 120 days treatment was reported to alleviate breast cancer in rats, increasing the expression of apoptotic proteins while suppressing expression of anti-apoptosis proteins [40]. Overall, it is well-described in the literature that the effectivity of Manuka honey against breast cancer is very specific, as the effects from previous studies do not represent the whole living organism. Furthermore, the existence of methylglyoxal in New Zealand honey highly contributed to the Unique Manuka Factor that was also claimed to be responsible for potent antibacterial activity against *Staphylococcus aureus*, especially in wound healing [46,47]. In the present study, methylglyoxal was also observed in honey from New Zealand based on ^1^H NMR spectra. However, the collected New Zealand samples only inhibited the growth of the tested cancer cell lines by 30% at most. New Zealand honey is constituted mainly of polar metabolites such as saccharides and low molecular weight lipids.

From the literature reports exemplified in this paper, there was quite a high expectation of a better result for the Manuka Honey samples. Manuka plants occur in both the north and south islands, as the collected Manuka honey samples were for this study. Phytochemical variations between the plants from the two geographical areas have been described [48,49], providing evidence that there are three chemotypes in New Zealand: (1) a high-pinene chemotype from the far north, (2) a high-triketone chemotype from East Cape, and (3) the widely distributed chemotype containing a complex of sesquiterpenes. The high-triketone samples from East Cape exhibited the most potent antimicrobial activity. Regarding anticancer activity, published papers to date did not specify a detailed source of the Manuka honey under investigation. As mentioned in the earlier section of this study, the multivariate analysis of the fused datasets by PCA and OPLS exhibited differences in chemical profiles between the North and South Island samples. The South Island samples were in combination with other plant sources such as clover, honey dew, and borage, while the North Island samples were collected as Manuka Blend. With the difference in product profile that reflects the clear variation between the two groups, bioactivity was anticipated for the North Island samples due to their declared high manuka content. However, no anti-oncogenic activity was observed from both groups. The question we currently post is how cell-specific the bioactivity of the Manuka honey or of a *Leptospermum* plant extract or its metabolites is. Currently, there are four papers published on *Leptospermum*, two of which are on the antimicrobial activities of its sesquiterpenoid [28] and flavonone [27] metabolites, while anticancer bioassays have only been performed on the Manuka honey samples against colon cancer HCT-116 [50] and on three other cancer cell lines, murine melanoma (B16.F1), colorectal carcinoma (CT26), and human breast cancer (MCF-7) [45]. However, the two latter papers did not present any chemical analysis of their samples. For our anticancer bioassays, we used adenocarcinoma human alveolar basal epithelial cell (A549), human ovarian cancer cell (A2780), human pancreatic cancer cell (PANC-1), and human breast cancer cell (ZR75). The work by Fernandez-Cabezudo et al. [45] exhibited a treatment with Manuka honey alone of about 33% inhibition of tumor growth that is quite comparable to our results on ZR75 for North Island samples (LNZ, MNZ, NNZ, ONZ, PNZ, QNZ, SNZ, TNZ, UNZ, VNZ, and WNZ). On the other hand, Fernandez-Cabezudo et al. did observe 61% inhibition in combination with paclitaxel. Similarly, it was also reported that the bioactivity 5-fluorouracil on HCT-116 was enhanced by Manuka honey [50].

## 4. Materials and Methods

### 4.1. Honey

Fresh Malaysian and New Zealand honeys were randomly obtained from several beekeepers from Malaysia and New Zealand (Supplementary Table S4). Samples were kept at room temperature until further use.

### 4.2. Extraction of Fresh Malaysia and New Zealand Honeys

Samples of honey were weighed at 1 g each and dissolved in 25 mL of 1:1 water and methanol by stirring for a minimum duration of 2 h. The supernatant was filtered and collected in a round-bottom flask and concentrated in vacuo on a rotary evaporator, R-110 and R-3 (BŪCHI Labortechnik AG, Flawil, Switzerland) at a standardised temperature of 40 °C. The extraction with aqueous methanol was repeated 3 times. Once dry, all the samples were transferred to 5 mL tared vials by reconstituting the samples in an appropriate solvent such as acetone, methanol or distilled water, or a combination of those solvents. Samples that were more difficult to reconstitute in solution were sonicated using Ultrawave sonicator (Scientific Laboratory Supplies, Ltd., Coatbridge, UK). The samples were then further concentrated on heating blocks (STUART Bibby Scientific Limited Stone, Staffordshire, UK) at 40 °C under a stream of nitrogen gas. Water-soluble extracts were lyophilised in the Christ Alpha 2–4 freeze dryer (Martin Christ Gefriertrocknungsanlangen GmbH, Osterode am Harz, Germany) at −80 °C. All the dried concentrated samples were weighed and stored in closed vials at room temperature until further analysis.

### 4.3. Liquid Chromatography High Resolution Mass Spectrometry (LC-HRMS) and Dereplication

All crude extracts were prepared to a concentration of 1 mg/mL in methanol (MeOH, HPLC-grade). A blank solvent was also included. A silica C-18 HPLC column with the size of 70 × 3.0 mm^2^, particle size of 5 µm and pore size of 100 °A (Hichrom Limited, Reading, UK) was used. The experiment was carried out according to an established standard operating procedure [13] using the ThermoFinnigan Exactive Orbitrap Mass Spectrometry (Thermo Fisher Scientific, GmbH, Bremen, Germany) in both positive and negative ionisation on switch mode. Please also see supplementary information for details. LC-MS data was recorded using Xcalibur version 2.2 (Thermo Fisher Scientific, GmbH Bremen, Germany). The LC-MS Xcalibur raw data from both positive and negative ionization modes were sliced using the MassConvert file converter to separate both positive and negative masses and processed using Mzmine 2 following earlier published protocol [13]. Please see Supplementary Information for details. Dereplication was done using an In-house EXCEL Macro as earlier described [13]. The EXCEL Macro file was coupled with the Dictionary of Natural Product (DNP) database for peak identification and dereplication. The data were then converted into a CSV file that was exported into SIMCA 17.0 (Umetrics, Umeå, Sweden) for multivariate analysis.

### 4.4. Nuclear Magnetic Resonance (NMR) Spectroscopy

All crude extracts were prepared at a concentration of 5 mg per 600 µL deuterated DMSO-d_6_ (Sigma-Aldrich, Dorset, UK). The ^1^H and COSY (400Hz) spectrums were run for 1-dimensional correlation spectroscopy NMR in JEOL-LA400 FT-NMR instrument equipped with a 40TH5AT/FG probe (JEOL, Tokyo, Japan). Each spectrum was accumulated with 64 scans, a delay time of 4 s, and an acquisition time of 3.41 min with 32 Kb. Prior to multivariate analysis, the data obtained were processed with MestReNova (Mnova10.0) software (Mestrelab Research, Santiago de Compostela, Spain). The ^1^H NMR spectrums were processed, which included Whittaker Smoother, manual phase correction and Gaussian set to 1 for apodization. Binning was applied at an integral region of 0.04 ppm using the Sum Method and normalised by largest peak at 1 × 10^2^. The processed spectral dataset was exported to EXCEL as a txt file then converted to csv file prior to importing to SIMCA for multivariate analysis.

### 4.5. Multivariate Analysis

The database generated from the EXCEL Macro was further analysed using SIMCA 17 for multivariate analysis. For the mass spectral data, the MZmine feature ID number was merged with ionization mode to generate a unique primary ID in SIMCA while the other variables such as retention time, *m*/*z*, and MW were considered secondary IDs. For the NMR data, the chemical shift in ppm is used to generate the unique primary ID and there are no secondary IDs considered for variables. The data were preliminarily analysed with an unsupervised statistical method by using principal component analysis (PCA). A supervised statistical analysis method was done with orthogonal partial least squares discriminant analysis (OPLS-DA) when comparing groups and discriminating metabolites according to the known variables between groupings. PCA and OPLS-DA analysis were done using Pareto scaling, and the models were validated based on multiple correlation coefficients (R^2^) and cross-validation (Q^2^) as well as permutation tests for the supervised method. HCA plots were done in SIMCA, calculated with single linkage and sorted by size. The discriminant data were cross-matched with the dereplication database through an in-house EXCEL Macro to pinpoint the putative metabolites for further isolation and purification. Heatmaps were generated using the programme R and loading package gplots.

### 4.6. Cytotoxicity Assay

Cytotoxicity assays were done using the Alamar Blue^TM^ assay [51] on adenocarcinoma human alveolar basal epithelial cell (A549), human ovarian cancer cell (A2780), human pancreatic cancer cell (PANC-1), human breast cancer cell (ZR75), and normal human foetal lung fibroblast (HFL-1). For the screening, the cytotoxicity assays for all extracts were performed at a concentration of 100 µg/mL. All extracts were tested in triplicate, and the viability percentages of control were calculated and measured. A cell viability percentage of ≤50% was classified as a positive screening result.

## 5. Conclusions

Based on the ZR75 assay, the honey extracts from Malaysia gave stronger cytotoxicity effects compared to New Zealand honey. In conclusion, the metabolite profiling of honey samples from Malaysia and New Zealand were best obtained employing both HR-LCMS and ^1^H NMR as analytical tools. The fused spectral dataset was a very useful tool in differentiating the honey samples according to their geographical sources and bioactivity across the subtle variations of their secondary metabolites, even at lower concentrations. Mild cytotoxicity on breast cancer cell line ZR75 was determined for CH, FH, GH, HH, JH, KH, LH, NH, RH, and SH. Nonetheless, all the bioactive extracts were giving protective effect on normal cell lines such as HFL-1. This is the first metabolomic profiling study that distinguished Malaysian and New Zealand honey for their potential as anticancer agents.

## Figures and Tables

**Table 1:** Bioassay results of honey samples at 100 µg/mL on cell viability of A549, A2780, ZR75, PANC-1 and HFL-1 as in percentage of the control, subjected to <50% threshold. In bold and numbers colour-coded in red represent positive cytotoxicity effects of sample extracts.

No.	ID	A549	A2780	ZR75	PANC-1	HFL-1	No.	ID	A549	A2780	ZR75	PANC-1	HFL-1
Malaysian Samples							New Zealand Samples						
**1**	AH	86	92	92	85	77	**20**	ANZ	90	79	68	89	87
**2**	BH	88	92	94	84	79	**21**	BNZ	89	93	66	88	86
**3**	**CH**	88	92	**38**	87	84	**22**	CNZ	83	90	93	86	78
**4**	DH	88	92	90	86	79	**23**	DNZ	85	91	92	83	71
**5**	EH	88	77	71	82	81	**24**	ENZ	86	86	92	88	82
**6**	**FH**	83	92	**41**	85	78	**25**	FNZ	90	92	69	87	81
**7**	**GH**	88	93	**41**	86	83	**26**	GNZ	84	92	92	88	82
**8**	**HH**	83	91	**39**	90	80	**27**	HNZ	88	93	90	88	81
**9**	IH	89	88	73	83	82	**28**	INZ	90	78	82	88	83
**10**	**JH**	87	93	**40**	85	81	**29**	JNZ	91	92	68	88	83
**11**	**KH**	84	92	**41**	83	78	**30**	KNZ	90	86	76	87	84
**12**	**LH**	85	92	**42**	85	80	**31**	LNZ	88	92	71	88	82
**13**	MH	87	92	70	84	74	**32**	MNZ	89	92	67	90	83
**14**	**NH**	87	93	**39**	89	84	**33**	NNZ	89	92	72	88	78
**15**	OH	85	92	73	86	79	**34**	ONZ	89	93	68	88	83
**16**	PH	90	91	72	86	81	**35**	PNZ	87	92	72	84	73
**17**	QH	87	93	77	80	80	**36**	QNZ	88	94	70	90	82
**18**	**RH**	89	83	**43**	89	83	**37**	RNZ	89	90	91	89	81
**19**	**SH**	85	75	**47**	88	83	**38**	SNZ	89	93	73	89	83
							**39**	TNZ	88	91	71	88	77
							**40**	UNZ	88	91	73	87	77
							**41**	VNZ	88	92	73	89	84
							**42**	WNZ	86	92	70	87	82

**Table 2:** Discriminating metabolites between Malaysian and New Zealand (NZ) honey samples extracted from the loading S-plots at *p* < 0.05. The list was arranged according to their *p* values as determined by OPLS-DA.

Mzmine ID ^a^	*m/z*	*p* Values ≤ 0.05	RT (min)	Predicted Molecular Formula (DBE) ^b^	Accurate Mass (∆ ppm)	Dereplicated Hits	Reported Source ^c^
**a.** **Malaysian samples** (**Bold** and *italicised* rows indicate metabolites bioactive against ZR75; highlighted rows were the discriminating metabolites also detected from the PCA-loadings plot.) Structures of dereplicated hit compounds are presented in Supplementary Figure S7a.							
N_1923 *	309.17 *	1.08 × 10^−4^	16.15	C_17_H_26_O_5_ (DBE = 5)	310.1776 (−1.370181)	gingerdiol 2-hexylphenol-O-β-D-xylopyranoside blumeaene L 9-acetyl-6,7-dihydroxy-3(15)-caryophyllen-8-one	*Zingiber officinale* *Leucas aspera* *Blumea balsamifera* *Buddleja davidii*
N_2025 *	321.21	1.41 × 10^−4^	29.19	C_16_H_34_O_4_S (DBE = 0)	322.2178 (0.055863)	cetyl sulphate	*Cocos nucifera*
N_2028 *	381.23	1.60 × 10^−4^	27.52	C_27_H_30_N_2_ (DBE = 14)	382.2392 (−4.442225)	no hits	
N_4469 *	311.17	1.77 × 10^−4^	20.46	C_10_H_20_N_10_O_2_ (DBE = 6)	312.1761 (−3.107211)	no hits	
N_4445 *	325.184	2.14 × 10^−4^	21.55	no prediction	326.1916	no hits	
P_3386 *	***295.13***	***3.11 × 10^−4^***	***19.25***	***C_19_H_18_O_3_*** **(DBE = 11)**	***294.1255*** ***(−0.322991)***	***Please see under 2c and structures shown*** ***in Figure 9e***	
P_4751 *	413.27	3.86 × 10^−4^	32.33	C_22_H_32_N_6_O_2_ (DBE = 10)	412.2590 (0.790766)	no hits	
N_4473 *	339.2	3.91 × 10^−4^	26.88	C_23_H_32_O_2_ (DBE = 8)	340.2401 (−0.382083)	plastoquinone 3	*Spinacia oleracea*
N_1976 *	337.21	6.68 × 10^−4^	37.35	no prediction	338.2128	no hits	
N_1925 *	353.20	9.43 × 10^−4^	21.38	C_24_H_32_O_2_ (DBE = 9)	352.2401 (−0.369066)	5-(12-phenyl-8-dodecenyl)resorcinol	*Knema laurina*
**b.** **NZ samples** (Highlighted rows were the discriminating metabolites also detected from the PCA-loadings plot). Structures of dereplicated hit compounds are presented in Supplementary Figure S7b.							
N_1928 *	397.227	1.65 × 10^−16^	22.19	C_27_H_30_N_2_O (DBE = 14)	398.2340 (−4.552579)	no hits	
N_7	195.05	2.39 × 10^−11^	1.33	C_6_H_12_O_7_ (DBE = 1)	196.0582 (−0.535555)	gluconic acid	*Aureobasidium pullulans*
N_720	215.03	3.76 × 10^−7^	1.28	C_16_H_6_O (DBE = 14)	214.0424 (2.499511)	no hits	
N_536	387.115	1.36 × 10^−7^	1.09	C_13_H_24_O_13_ (DBE = 2)	388.1218 (0.270534)	disaccharide (no hits in DNP)	
N_1011	225.06	1.55 × 10^−3^	1.34	C_7_H_14_O_8_ (DBE = 1)	226.0688 (−0.309640)	no hits	
N_723	161.05	5.40 × 10^−3^	1.34	C_6_H_10_O_5_ (DBE = 2)	162.0528 (−0.148100)	1,5-anhydrofructose	various plant sources
N_30	207.05	2.85 × 10^−2^	1.58	C_14_H_6_O_2_ (DBE = 12)	206.0370 (1.067771)	no hits	
N_4692	179.06	5.49 × 10^−2^	1.33	C_6_H_12_O_6_ (DBE = 1)	180.0634 (0.055536)	various hexoses e.g., fructose, allose	various plant sources *Protea rubropilosa*
**c.** **Predicted bioactive metabolites** (**Bold** and *italicised* rows indicate discriminating metabolites for Malaysian honey samples. Highlighted rows represent the metabolites with VIP values > 1.0.) Structures shown in Figure 9e.							
N_539	308.099	1.11 × 10^−4^	1.46	C_11_H_19_NO_9_ (DBE = 3)	309.1058 (−0.595265)	*O*-sialic acid (**1**)	enzymatic hydrolysis of sialoproteins and oligosaccharides
N_5017	321.210	8.28 × 10^−3^	37.32	C_16_H_34_O_4_S (DBE = 0)	322.2177 (−0.254486)	cetyl sulphate (**2**)	*Cocos nucifera*
N_1030	470.151	1.72 × 10^−2^	1.15	C_30_H_21_N_3_O_3_ (DBE = 22)	471.1587 (0.868073)	no hits	
P_5294	429.261	1.80 × 10^−2^	27.11	C_22_H_32_N_6_O_3_ (DBE = 10)	428.2539 (0.726205)	no hits	
N_5083	441.253	1.80 × 10^−2^	37.23	C_20_H_42_O_8_S (DBE = 0)	442.2602 (0.357256)	no hits	
P_3154	226.180	2.76 × 10^−2^	26.94	C_13_H_23_NO_2_ (DBE = 3)	225.1728 (−0.350842)	tropine isovalerate(**3**)	*Bruguieras exangula*
P_6857	355.282	3.10 × 10^−2^	26.54	C_17_H_34_N_6_O_2_ (DBE = 4)	354.2745 (0.496790)	no hits	
P_151	226.216	3.17×10^−2^	26.39	C_14_H_27_NO (DBE = 2)	225.2092 (−0.284180)	plumerinine (**4**)	*Plumeria rubra*
N_41	369.103	3.53 × 10^−2^	1.11	C_26_H_14_N_2_O (DBE = 21)	370.1108 (0.505254)	no hits	
P_1365	347.096	3.54 × 10^−2^	1.09	C_14_H_18_O_10_ (DBE = 10)	346.0883 (−4.912017)	tetra-acyl- mannolactone(**5**) tetra-acyl- ribonic lactone (**6**)	various plant sources
***P_3386***	295.133*	***3.75*** ***× 10^−2^***	***19.25***	***C_19_H_18_O_3_*** (DBE = 11)	***294.1255*** ***(−0.322991)***	***dianisylidene acetone (7)*** ***5-dehydroxy*** ***artocarbene (8)*** ***eupomatenoid 13 (9)*** ***methyloroxyl-opterocarpan(10)*** ***sterequinone H(11)***	***Curcuma longa*** ***Artocarpus incises*** ***Caryodaphnopsis tonkinensis*** ***Oroxylum indicum*** ***Stereospermum personatum***
P_6818	359.240	4.96 × 10^−2^	26.78	C_15_H_30_N_6_O_4_ (DBE = 4)	358.233 (0.407556)	no hits	
N_2122	279.164	5.06 × 10^−2^	20.35	C_13_H_28_O_4_S (DBE = 0)	280.1709 (0.242709)	no hits	

^a^ MZMine ID includes ionisation polarity: P-positive mode; N-negative mode, ^b^ DBE = Double Bond Equivalence, ^c^ Reported source is filtered to geographical origin of collected samples, * Ion peaks found as discriminating features for samples collected from South Island (NZ-south) with *p* < 0.05.

## Data Availability

All the information related to the study protocol and its results is available in the repository of the University of Strathclyde, included in the PhD Degree work entitled: “The Use of Metabolomics Tools to Assess the Therapeutic Natural Products of Honey and Propolis from Malaysia and New Zealand”. Yusnaini Binti MD Yusoff. 2018.

## Supplementary Materials

The following supporting information can be downloaded at: https://www.mdpi.com/article/10.3390/metabo12010085/s1, Table S1: Malaysia and New Zealand honey samples used in this study; Table S2: ^1^H NMR spectra of the main constituents found in collected honey samples in comparison to those described in the literature; Table S3: Summary of the number of features detected in the bioactive honey extracts: (a) total number of features in positive and negative ionization modes after the removal of features from solvent (blank) with intensity >1 × 10^5^; and (b) total number of features putatively identified by dereplication from the DNP database with number of unknowns; Table S4: Major metabolites found in honey samples in the positive ionisation mode; Figure S1: The Malaysian honey extracts ^1^H NMR spectra (400 MHz) in DMSO-d6; Figure S2: The New Zealand honey extracts ^1^H NMR spectra (400 MHz) in DMSO-d6; Figure S3: Dehydration of glucose to 5-hydroxymethyl furfural then by oxidation to its acid form (Lee, Harris, Champagne, & Jessop, 2016); Figure S4: (a) 1H and (b) COSY NMR (400 MHz) spectra of BNZ from New Zealand honey, indicating the presence of glucose moiety (red lines) and 5-(hydroxymethyl)furan-2-carboxylic acid (blue lines); Figure S5: Base peak chromatograms of Malaysia (MAS) and New Zealand (NZ) honey extracts in the positive (a and c) and negative (b and d) modes, respectively; Figure S6: Mass spectrum for NH in the positive ionization mode showing the presence of a cluster of features (a) within the RT range of 0.69–2.27 min; (b) within the RT range of 0.77–2.01 min; Figure S7: Structures of some dereplicated metabolites (a) found in Malaysian (MAS) honey samples; (b) found in New Zealand (NZ) honey samples.
