# Supplementary material for: Metabolomic Profiling of Malaysian and New Zealand Honey Using Concatenated NMR and HRMS Datasets
Source: Metabolites. 2022 Jan 17;12(1):85. doi: 10.3390/metabo12010085 (PMC8781004; doi:10.3390/metabo12010085)
Supplement: Supplementary file 1 [file metabolites-12-00085-s001.zip › Supplementary information-ree3 (1).pdf]

Metabolomic profiling of Malaysian and New Zealand Honey using concatenated NMR and HRMS datasets

Yusnaini M. Yusoff [a], Grainne Abbott [a], Louise Young [a], RuAngelie Edrada-Ebel [a*]

[a] Strathclyde Institute of Pharmacy and Biomedical Sciences
University of Strathclyde, The John Arbuthnott Building
161 Cathedral Street, Glasgow G4 0RE, UK
*Email: ruangelie.edrada-ebel@strath.ac.uk Tel: +44(0)1415485968
Fax: +44(0)1415522562

Mass Spectrometry

Methanol (MeOH), dichloromethane (DCM), acetonitrile (MeCN) and formic acid were purchased (Fisher Scientific, Hemel Hempstead, UK). All reagents were of analytical grade. HPLC grade water was obtained in-house from a direct Q-3 water purification system (Millipore, Watford, UK). Samples and medium control samples were prepared at a concentration of 1 mg/mL in 80:20 MeOH: DCM. A solvent blank was also included. Experiments were carried out using an Exactive mass spectrometer with an electrospray ionization source attached to an Accela 600 HPLC pump with Accela autosampler and UV/Vis detector (Thermo Scientific, Bremen, Germany). The mass accuracy was set to less than 3.0 ppm. The Orbitrap mass analyzer can limit the mass error within ±3.0 ppm. The instrument was calibrated to maintain a mass accuracy of ±1.0 ppm by applying the lock mass function. The instrument was externally calibrated according to the manufacturer's instructions before the run and was internally calibrated during the run using lock masses. In positive ion mode, lock masses were *m/z* 83.06037 (acetonitrile dimer) and *m/z* 195.08625 (caffeine) and in negative ion mode the lock mass was *m/z* 91.00368 (formic acid dimer). Mass spectrometry was carried out over a mass range of 100–2000 *m/z* in positive and negative ionization modes with spray voltage of 4.5 kV and capillary temperature at 270 °C. Ten µL was injected from each vial, at a flow rate of 300 µL/min. The column used was an ACE5 C18 column (5 µm ×75 mm ×3 mm) (Hichrom Limited, Reading, UK). A binary gradient method was utilized. The two solvents were A (water and 0.1% formic acid) and B (MeCN and 0.1% formic acid). The gradient was carried out for 45 minutes and the program followed; at zero minutes A = 90% and B = 10%, at 30 min A = 0% and B = 100% at 36 min A = 90% and B = 10% until end at 45 min. The UV absorption wavelength was set at 254 nm, the sample tray temperature was maintained at 4 °C and the column maintained at 20 °C. The samples were run sequentially, with solvent and media blanks analyzed first. LC-MS data was acquired using Xcalibur version 2.2 (Thermo Scientific, Bremen, Germany).

LC-MS data analysis using Mzmine 2 adapted from Macintyre et al., (2014)

The LC-MS Xcalibur raw data from both positive and negative ionization modes were sliced using the MassConvert file converter to separate both positive and negative masses. The mass files were uploaded separately and processed using Mzmine 2.10 software. The raw data methods were processed using peak detection, started with mass detection by mass detector was set as centroid, noise level at 1000 and MS level as 1. Followed by chromatogram builder was set at 0.2 min for Min time span, Min height at 10000 and *m/z* tolerance at 0.001 *m/z* or 5 ppm. The analysis continued with peak detection from peak list methods, for the chromatogram deconvolution, where the algorithm was set as local minimum search. The chromatographic threshold was set to 5%, search minimum in RT range at 0.4 min, minimum relative height at 5%, minimum absolute height at 10000, Min ratio of peak top/edge as 3 and peak duration range within 0.2 – 5 min. Meanwhile for deisotope, *m/z* tolerance was set to 0.001 *m/z* or 5 ppm, retention time tolerance at 0.1 min absolute, maximum charge as 2 and representative isotope was set as most intense. For alignment, join aligner was set by following setting, including m/z tolerance at 0.001 *m/z* or 5 ppm, retention time tolerance was set to 5% relative, weight for RT and m/z at 20 because those parameters are given equal importance.

Followed by gap filling analysis, peak finder was set as following parameters, *m/z* tolerance at 0.001 *m/z* or 5 ppm, intensity tolerance at least 30% and retention time tolerance at 0.5 min absolute with RT correction. An adduct search for peak identification was analysed for RT tolerance at 0.2 min absolute, adducts for positive mode was set as Na, K, NH4 and ACN + H, *m/z* tolerance at 0.001 *m/z* or 5 ppm and maximum relative adduct peak height at 30%. The complex search was also done by following parameters; ionization was set as M+H for positive mode, retention time tolerance at 0.2 min absolute, *m/z* tolerance at 0.001 *m/z* or 5 ppm and maximum complex peak height at 50%. Lastly, the formula prediction was done by setting the charge at 1, ionization as [M+H]+, *m/z* tolerance at 0.001 *m/z* or 5 ppm and isotope pattern filter was done with all features with isotope peaks. The data was exported as CSV file until further clean-up process. All the Mzmine processing steps were then repeated with negative mode with some modification such as an adduct search was set as formate and ACN + H, complex search ionization was set as M−H and formula prediction ionization was set as $[M-H]^-$.

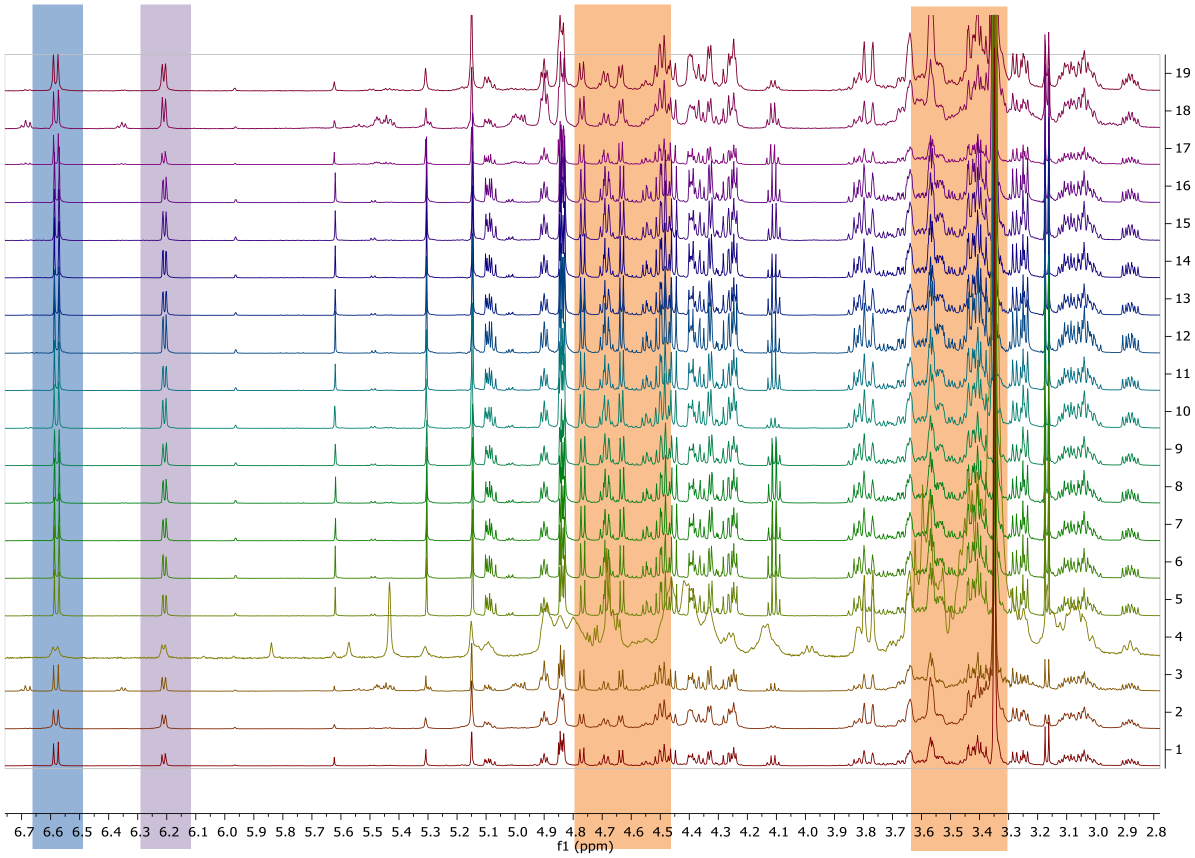

Figure S1: The Malaysian honey extracts ^{1}H NMR spectra (400 MHz) in DMSO-d_6. The regions within 3.51 – 4.70 ppm were determined for fructose overlapping with glucose (highlighted in orange), the anomeric proton of glucose at 6.58 ppm (highlighted in blue), and 5-hydroxymethyl-furan-2-carboxylic acid at 6.21 ppm (highlighted in purple). The spectra were labelled from 1 to 19 to represent sample codes MH, NH, OH, PH, AH, BH, CH, DH, EH, FH, GH, HH, IH, JH, KH, LH, QH, RH and SH, respectively.

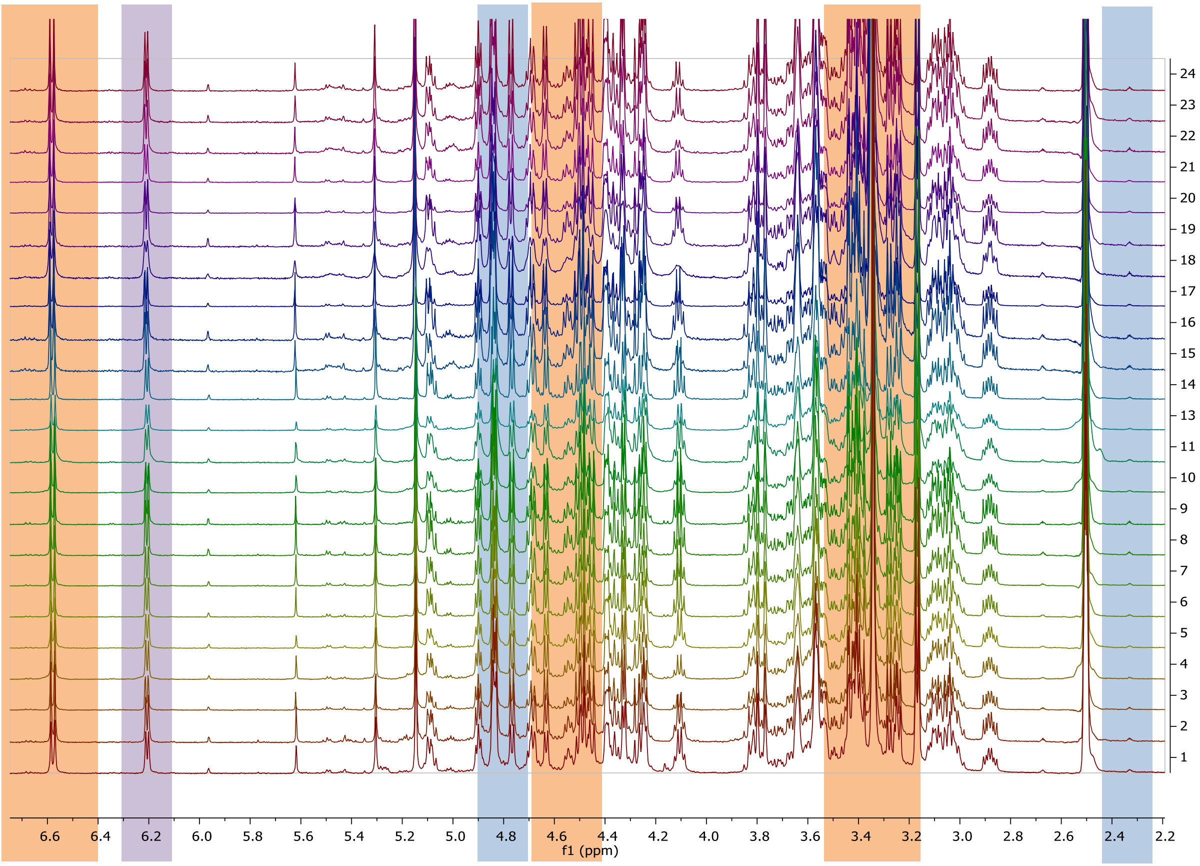

Figure S2: The New Zealand honey extracts ^{1}H NMR spectra (400 MHz) in DMSO-d_6. The signal at 2.31 and 4.81 ppm was assigned respectively for methyl and alkyl proton in methylglyoxal (highlighted in blue). The spectrum within 3.51 – 6.58 ppm were determined for glucose and fructose (highlighted in orange) and the signal at 6.21 ppm is the prediction of 5-HMF (highlighted in purple). The spectra were labelled from 1 to 23 to represent sample codes KNZ, LNZ, MNZ, NNZ, ONZ, PNZ, QNZ, RNZ, SNZ, TNZ, UNZ, VNZ, WNZ, ANZ, BNZ, CNZ, DNZ, ENZ, FNZ, GNZ, HNZ, INZ and JNZ, respectively.

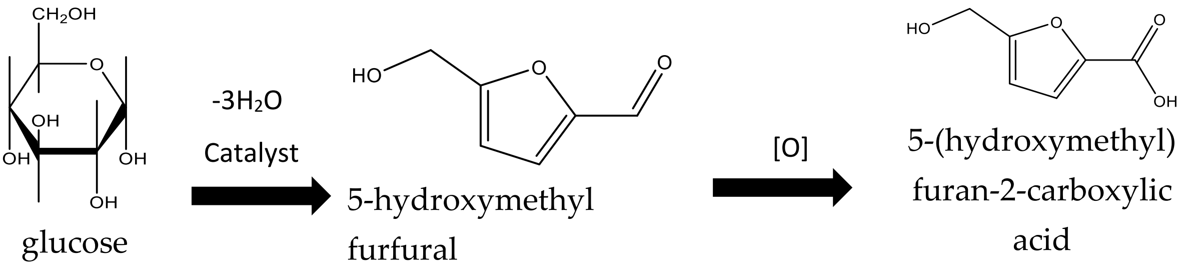

Figure S3: Dehydration of glucose to 5-hydroxymethyl furfural then by oxidation to its acid form (Lee, Harris, Champagne, & Jessop, 2016).

Table S1: ^{1}H NMR spectra of the main constituents found in collected honey samples in comparison to those described in the literature.

Compound name	Collected honey samples (DMSO-*d*$_6$)	Brazilian honey (D_2O) (Boffo, Tavares, Tobias, Ferreira, & Ferreira, 2012)	(Ohmenhaeuser, Monakhova, Kuballa, & Lachenmeier, 2013)*	(Spiteri et al., 2015)*
hydroxymethylfurfural	-	-	9.43–9.47 (*s*)	6.69, 7.54
(hydroxymethyl)furan-2-carboxylic acid	6.21, 4.89	-	-	-
formic acid	-	8.45 (s)	8.44–8.47 (*s*)	-
phthalic acid	-	-	7.53–7.48 (*m*)	-
fumaric acid	-	-	6.53–6.55 (*s*)	-
pyruvic acid	-	-	6.42–6.45 (*s*)	-
L(+)-rhamnose	5.00-5.25	-	5.13–5.09 (*d*)	-
glucose	6.59, 4.24-4.89	3.23-5.22	4.63–4.65 (*s*)	4.65, 3.51
arabinose	4.50-4.75	-	4.52–4.54 (*s*)	-
L(+)-tartaric acid	4.24-4.50	-	4.32–4.35 (*s*)	-
fructose	3.50-4.00	3.52-4.10	4.14–4.08 (*d*)	4.04, 3.58
sucrose	4.24-5.50	-	-	5.41, 4.20
malic acid	2.75-3.00	-	2.73–2.70 (*s*)	-
citric acid	-	2.79-2.94	2.69–2.68 (*s*)	-
succinic acid	2.50	-	2.50–2.52 (*s*)	-
methylglyoxal	2.31, 4.81	-	-	1.38, 2.31

*NMR buffer (KH_2PO_4, NaN_3), distilled water, NMR lock solution (trimethylsilyl propionate, D_2O)

a)

b)

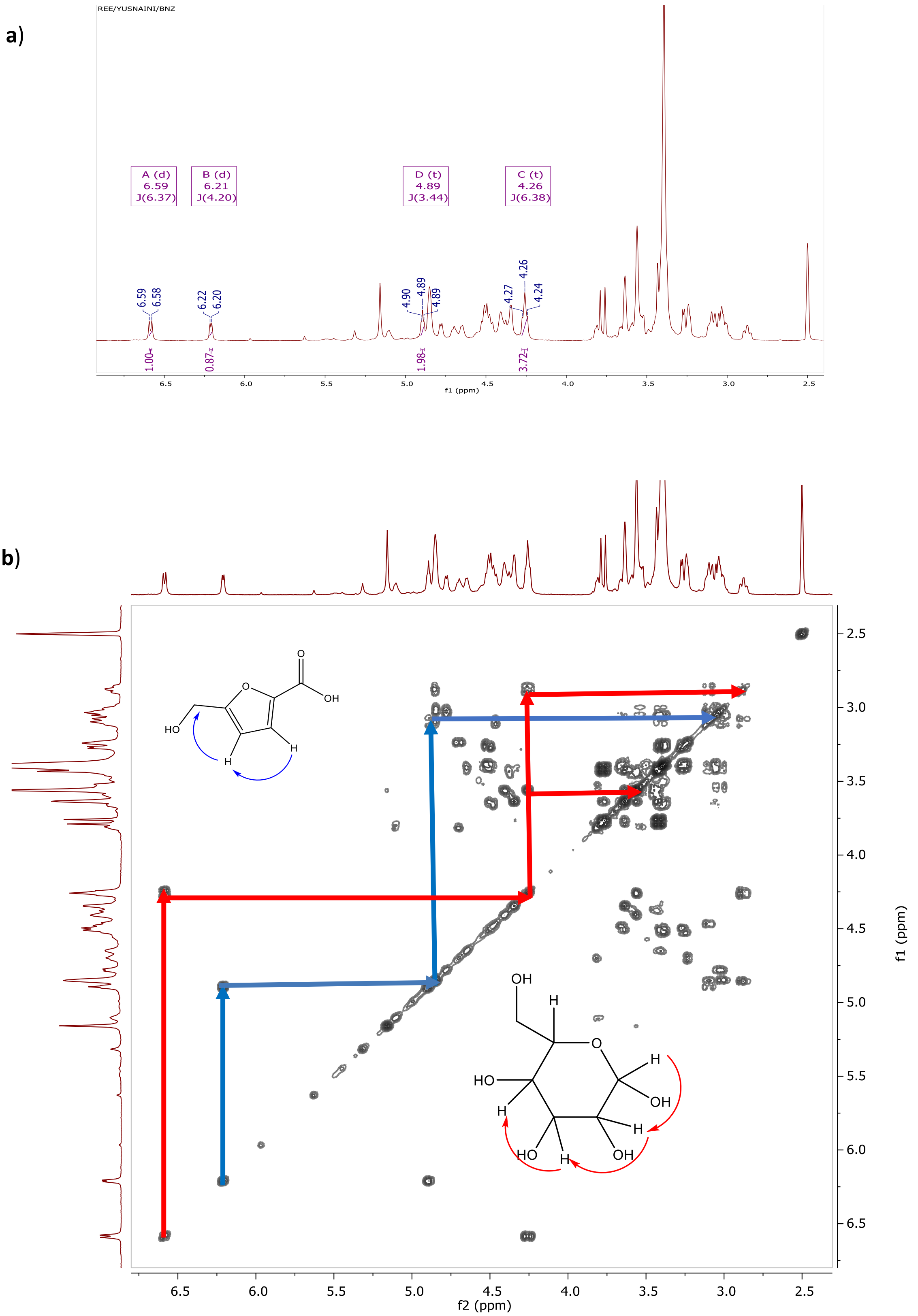

Figure S4: **a**) ^{1}H and **b**) COSY NMR (400 MHz) spectra of BNZ from New Zealand honey, indicating the presence of glucose moiety (red lines) and 5-(hydroxymethyl)furan-2-carboxylic acid (blue lines).

Figure S5: Base peak chromatograms of Malaysia (MAS) and New Zealand (NZ) honey extracts in the positive (**a** and **c**) and negative (**b** and **d**) modes, respectively.

a)

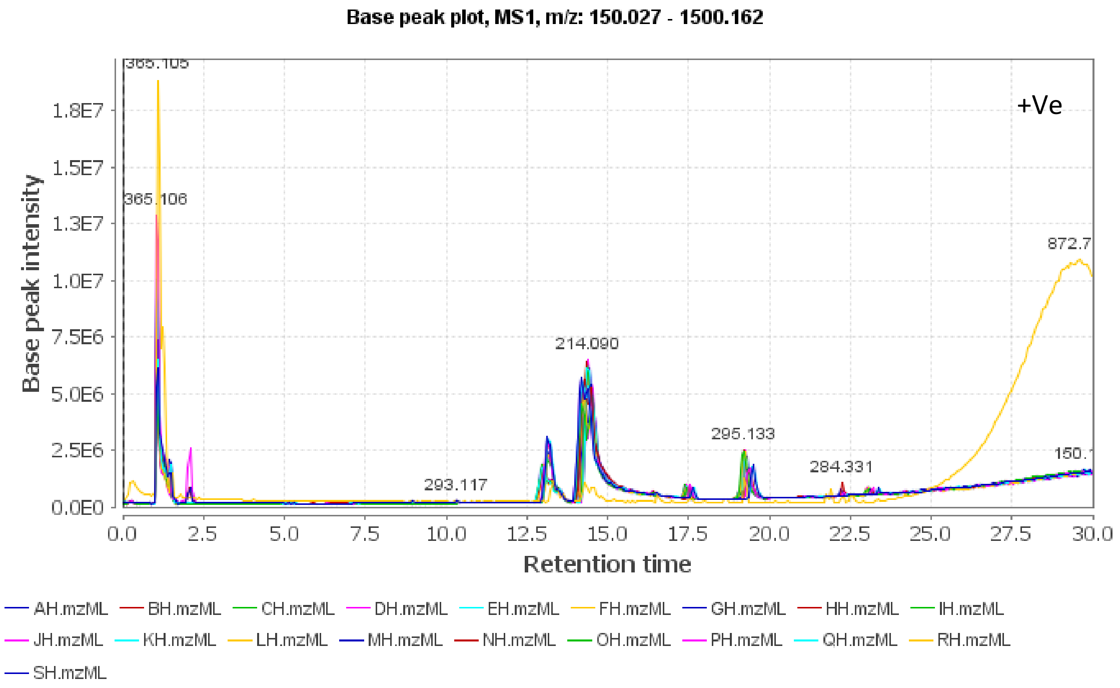

b)

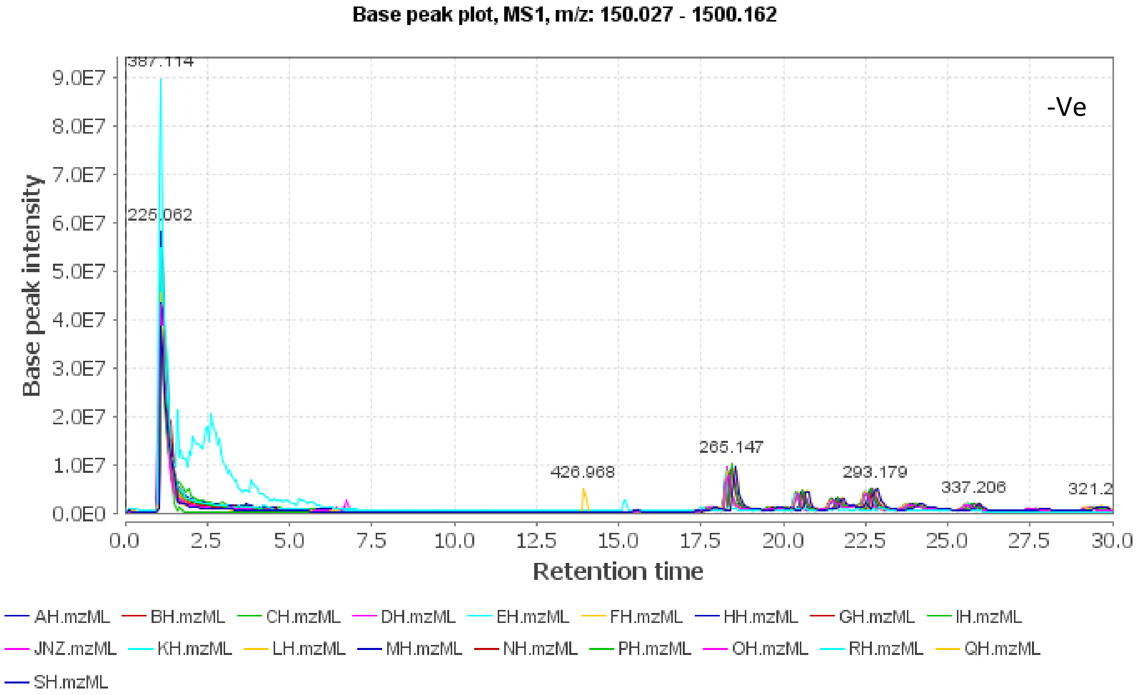

c)

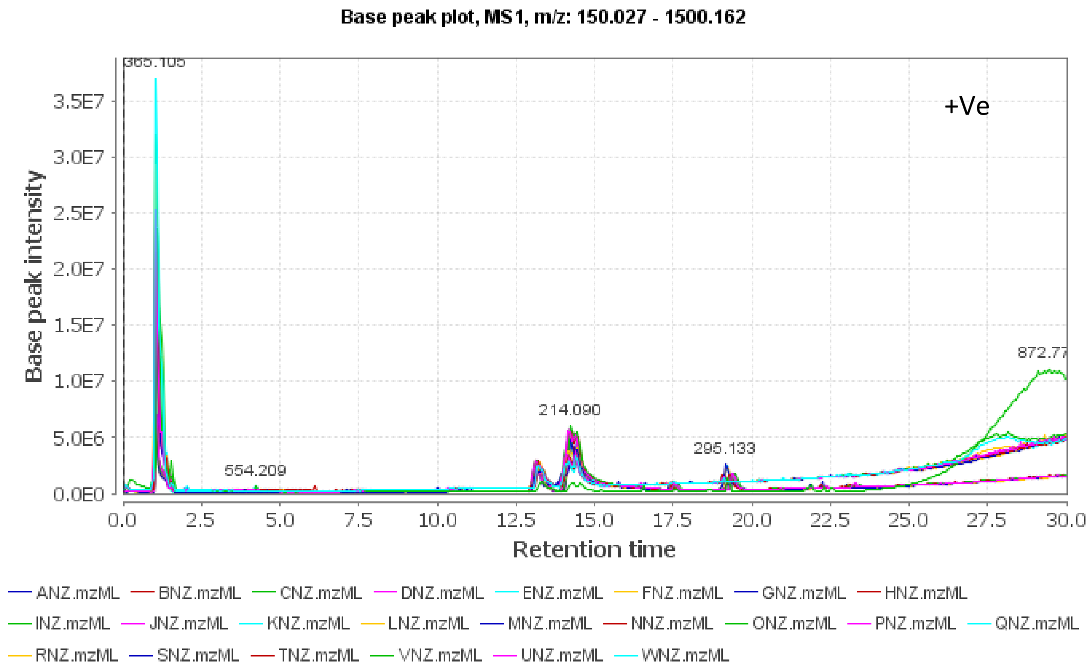
Base peak plot, MS1, m/z: 150.027 - 1500.162
+Ve
Base peak intensity
Retention time
365.105
554.209
214.090
295.133
872.77
3.5E7
3.0E7
2.5E7
2.0E7
1.5E7
1.0E7
5.0E6
0.0E0
0.0
2.5
5.0
7.5
10.0
12.5
15.0
17.5
20.0
22.5
25.0
27.5
30.0
ANZ.mzML BNZ.mzML CNZ.mzML DNZ.mzML ENZ.mzML FNZ.mzML GNZ.mzML HNZ.mzML
INZ.mzML JNZ.mzML KNZ.mzML LNZ.mzML MNZ.mzML NNZ.mzML ONZ.mzML PNZ.mzML QNZ.mzML
RNZ.mzML SNZ.mzML TNZ.mzML VNZ.mzML UNZ.mzML WNZ.mzML

d)

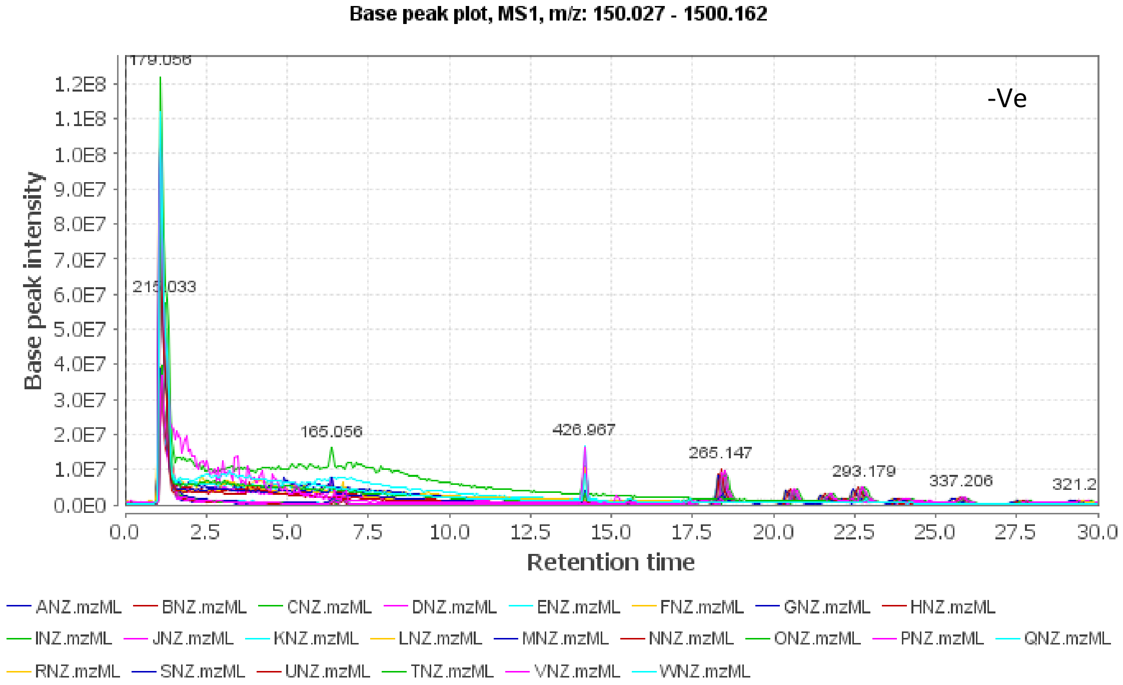
Base peak plot, MS1, m/z: 150.027 - 1500.162
-Ve
Base peak intensity
Retention time
179.056
215.033
165.056
426.967
265.147
293.179
337.206
321.2
1.2E8
1.1E8
1.0E8
9.0E7
8.0E7
7.0E7
6.0E7
5.0E7
4.0E7
3.0E7
2.0E7
1.0E7
0.0E0
0.0
2.5
5.0
7.5
10.0
12.5
15.0
17.5
20.0
22.5
25.0
27.5
30.0
ANZ.mzML BNZ.mzML CNZ.mzML DNZ.mzML ENZ.mzML FNZ.mzML GNZ.mzML HNZ.mzML
INZ.mzML JNZ.mzML KNZ.mzML LNZ.mzML MNZ.mzML NNZ.mzML ONZ.mzML PNZ.mzML QNZ.mzML
RNZ.mzML SNZ.mzML UNZ.mzML TNZ.mzML VNZ.mzML WNZ.mzML

Table S2: Summary of the number of features detected in the bioactive honey extracts: (a) total number of features in positive and negative ionization modes after the removal of features from solvent (blank) with intensity >1 × 10^5; and (b) total number of features putatively identified by dereplication from the DNP database with number of unknowns.

Bioactive honey extracts	(a) Total number of features (*m/z*)		(b) Total number of features identified by dereplication with DNP	
	Positive ion mode	Negative ion mode	Putatively identified in positive and negative modes	Unidentified in positive and negative modes
CH	2638	2034	1354 (29.0%)	3303 (71.0%)
FH	2620	2009	1329 (28.8%)	3286 (71.2%)
GH	2587	2003	1317 (28.8%)	3258 (71.2%)
HH	2591	1999	1331 (29.0%)	3244 (71.0%)
JH	2585	1975	1315 (28.9%)	3231 (71.1%)
KH	2392	2038	1324 (30.0%)	3094 (70.0%)
LH	2646	2020	1330 (28.6%)	3321 (71.4%)
MH	2680	2114	1355 (28.3%)	3426 (71.7%)
NH	2743	2247	1383 (27.8%)	3593 (72.2%)
RH	2138	1870	1162 (29.1%)	2831 (70.9%)
SH	2656	1741	1317 (30.0%)	3068 (70.0%)

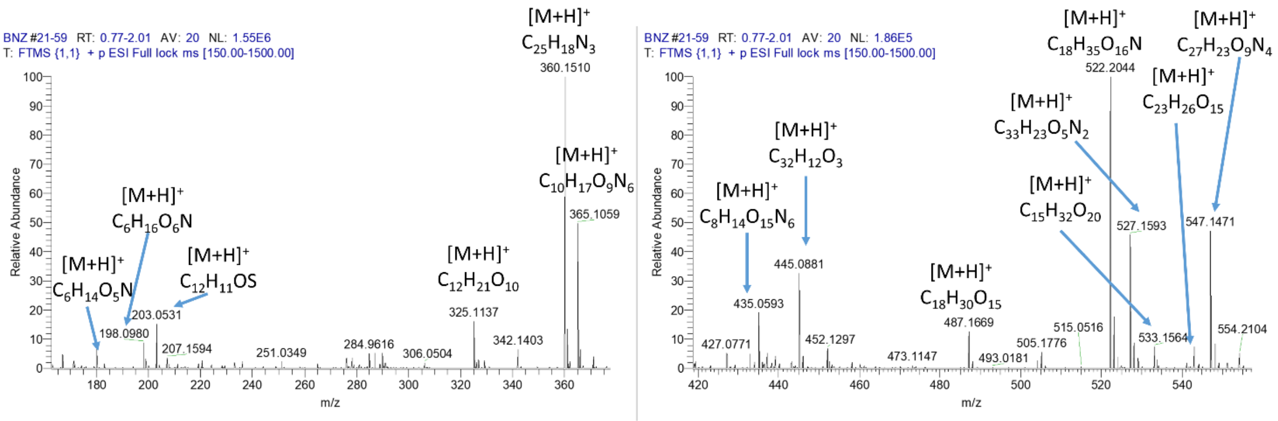

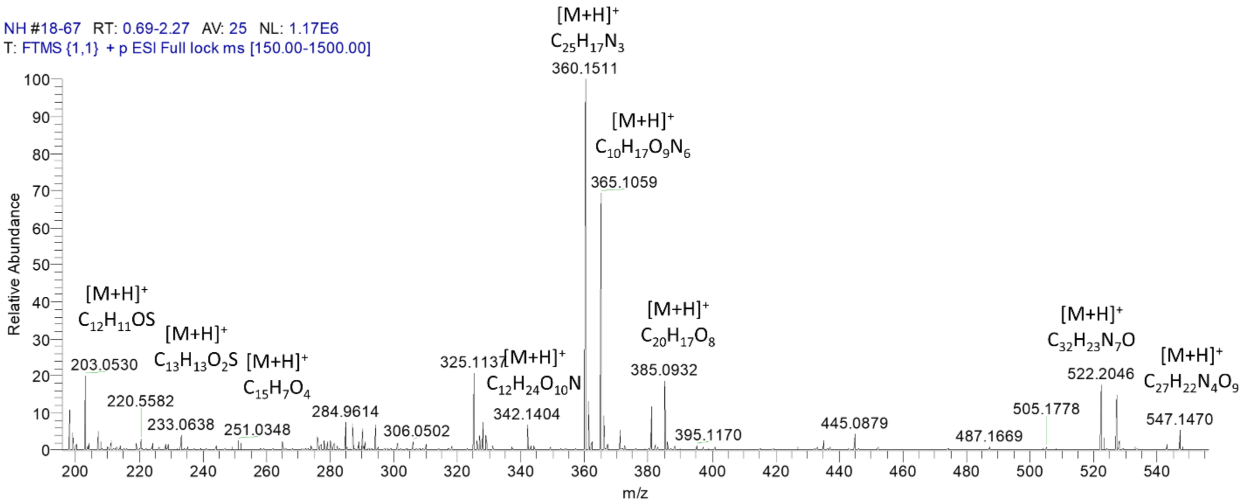

Figure S6 (a). Mass spectrum for NH in the positive ionization mode showing the presence of a cluster of features within the RT range of 0.69–2.27 min.. (**b**). Mass spectrum for BNZ in the positive ionization mode showing the presence of a cluster of features within the RT range of 0.77–2.01 min. Dereplicated ion peaks are presented on Table S4.

Table S3. Major metabolites found in honey samples in the positive ionisation mode. All these metabolites were also detected in negative ionization mode. Structures are presented in Figure. S7.

m/z	Rt	MF (DBE)	Accurate Mass (□ ppm)	DNP hits	Source
**Malaysian (MAS) samples**					
203.0526	1.07	$C_{12}H_{10}OS$ (DBE=8)	202.0453 (0.311811)	1-(2,3-dihydro-2-furyl)-4-(thien-2-yl)but-1-en-3-yne	*Chrysanthemum macrotum*
365.1055	1.07	$C_{18}H_{20}O_6S$ (DBE=9) $C_{10}H_{16}N_6O_9$ (DBE=6)	364.0982 (0.379019) (0.881631)	no hits	
342.1397	1.10	$C_{12}H_{23}NO_{10}$ (DBE=2)	341.1325 (0.882356)	2-amino-2-deoxy-3-glucopyranosylgalactose	
522.2036	1.10	$C_{32}H_{23}N_7O$ (DBE=25)	521.1963 (-0.207216)	no hits	
360.1501	1.11	$C_{25}H_{17}N_3$ (DBE=19)	359.1428 (1.539780)	bisindolizinylquinoline	
547.1466	1.19	$C_{27}H_{22}N_4O_9$ (DBE=19)	546.1393 (1.133412)	no hits	
385.359.1 4280923[a]	1.28	$C_{20}H_{16}O_8$ (DBE=13)	384.0851 (1.510084)	vitelignin A	*Vitex negundo*
233.0633	1.34	$C_{13}H_{12}O_2S$ (DBE=8)	232.0560 (0.853243)	5-[5-(methylthio)-4-penten-2-ynyl]-2-furanacrolein	*Chrysanthemum coronarium*
251.0342	1.38	$C_{15}H_6O_4$ (DBE=13)	250.0269 (1.159877)	no hits	
**New Zealand (NZ) samples**					
527.1193	1.05	$C_{33}H_{22}N_2O_5$ (DBE=24)	526.1518 (-2.039331)	no hits	
543.1332[b]	1.06	$C_{23}H_{26}O_{15}$ (DBE=11)	542.1260 (-2.167388)	5,6-diglucopyranosylangelicin	*Ficus ruficaulis* var. *antaoensis*
203.0526	1.07	$C_{12}H_{10}OS$ (DBE=8)	202.0453 (0.311811)	1-(2,3-dihydro-2-furyl)-4-(thien-2-yl)but-1-en-3-yne	*Chrysanthemum macrotum*
365.1055	1.07	$C_{18}H_{20}O_6S$ (DBE=9) $C_{10}H_{16}N_6O_9$ (DBE=6)	364.0982 (0.379019)	no hits	
325.1137	1.09	$C_{12}H_{20}O_{10}$ (DBE=3)	324.1058 (0.465897)	maltosan	
487.1665	1.10	$C_{18}H_{30}O_{15}$ (DBE=4)	486.1592 (1.491283)	galactopyranuronosyl-rhamnopyranosyl-rhamnose	*Hydrangea paniculata*
522.2036	1.10	$C_{18}H_{35}NO_{16}$ (DBE=25)	521.1963 (1.366090)	no hits	
360.1501	1.11	$C_{25}H_{17}N_3$ (DBE=19)	359.1428 (1.539780)	bisindolizinylquinoline	
533.1562	1.17	$C_{15}H_{32}O_{20}$ (DBE=0)	532.1489 (0.375835)	no hits	
547.1466	1.19	$C_{27}H_{22}N_4O_9$ (DBE=19)	546.1393 (1.133412)	no hits	
180.0867	1.26	$C_6H_{13}NO_5$ (DBE=1)	179.0794 (0.145187)	6-amino-1,2,3,4,5-cyclohexanepentol	
445.0871	1.39	$C_{32}H_{12}O_3$ (DBE=27)	444.0798 (2.600891)	no hits	
435.0580	1.40	$C_8H_{14}N_6O_{15}$ (DBE=5)	434.0507 (-2.347647)	no hits	
198.0973	1.51	$C_6H_{15}NO_6$ (DBE=0)	197.0901 (0.816886)	no hits	

[a] Only found in NH. [b] Only found in BNZ.

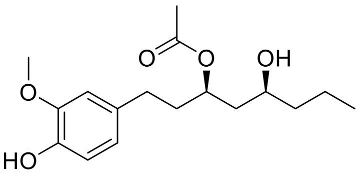

gingerdiol

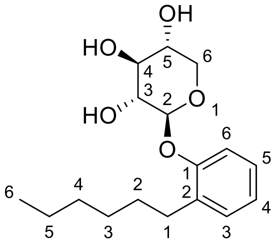

2-hexylphenol-O-β-D-xylopyranoside

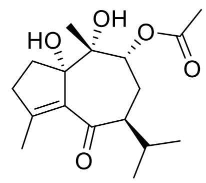

blumeaene L

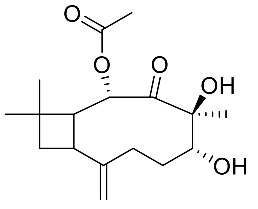

9-acetyl-6,7-dihydroxy-3(15)-caryophyllen-8-one

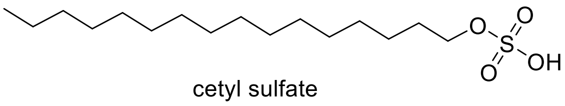

cetyl sulfate

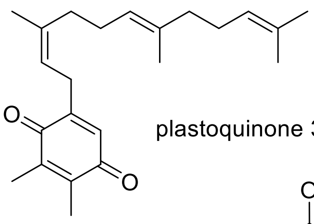

plastoquinone 3

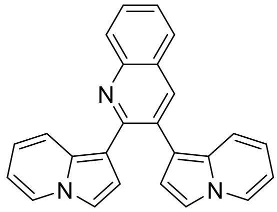

bisindolizinylquinoline

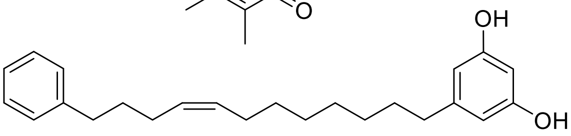

5-(12-phenyl-8-dodecenyl)resorcinol

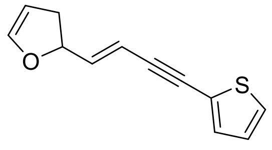

1-(2,3-dihydro-2-furyl)-4-(thien-2-yl)but-1-en-3-yne

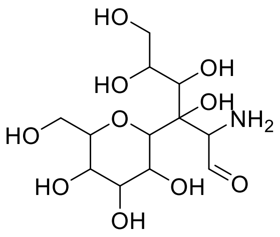

2-amino-2-deoxy-3-glucopyranosylgalactose

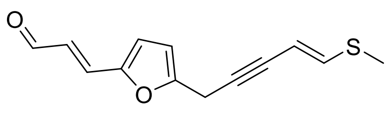

5-[5-(methylthio)-4-penten-2-ynyl]-2-furanacrolein

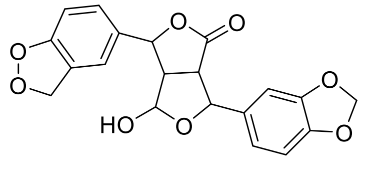

vitelignin A

Figure S7 (a). Structures of some dereplicated metabolites found in Malaysian (MAS) honey samples.

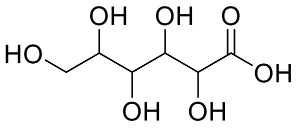

gluconic acid

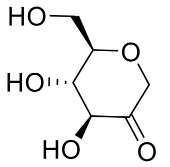

1,5-anhydrofructose

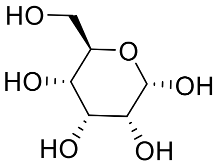

allose

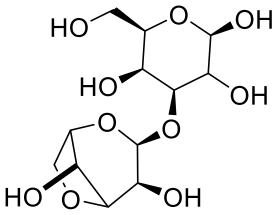

maltosan

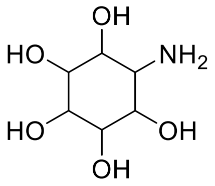

6-amino-1,2,3,4,5-cyclohexanepentol

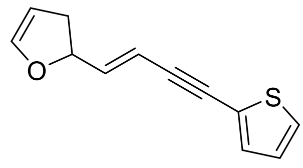

1-(2,3-dihydro-2-furyl)-4-(thien-2-yl)but-1-en-3-yne

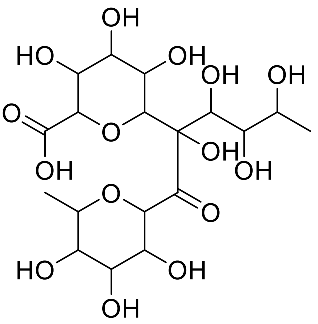

galactopyranuronosyl-rhamnopyranosyl-rhamnose

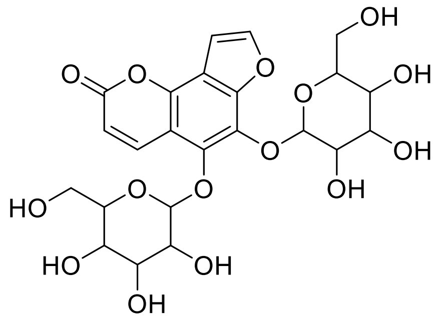

5,6-diglucopyranosylangelicin

Figure S7 (b). Structures of some dereplicated metabolites found in New Zealand (NZ) honey samples.

Table S4: Malaysia and New Zealand honey samples used in this study.

no.	ID	SOURCE OF NECTAR	TYPE OF BEE	GEOGRAPHICAL AREA	DISTRIBUTOR
			**MALAYSIA**		
**1**	AH	*Hevea brasiliensis*	*Apis mellifera*	Rubber tree estate, Johor, Malaysia	Beeshop (Mrs Cathie)
**2**	BH	*Cinnamomum cassia*	*Apis cerana*	Hilly area, Malaysia	
**3**	CH	Acacia tree species	*Apis trigona*	Negeri Sembilan, Malaysia	
**4**	DH	*Melaleuca*	*Apis mellifera*	Malacca, Malaysia	
**5**	EH	Multifloral	*Apis cerana*	Malaysia	
**6**	FH	*Koompassia excels*	*Apis dorsata*	Malaysia	
**7**	GH	*Momordica charantia*	*Apis mellifera*	Pahang, Malaysia	
**8**	HH	*Melaleuca*		Hilly area, Malaysia	
**9**	IH	Multifloral	*Apis trigona*	Negeri Sembilan, Malaysia	
**10**	JH	*Asystasia gangetica*	*Apis mellifera*	Malaysia	
**11**	KH	Multifloral (fruity tree)	*Geniotrigona thoracica*	Negeri Sembilan, Malaysia	
**12**	LH	*Acacia mangium*	*Apis mellifera*	Negeri Sembilan, Malaysia	
**13**	MH	*Ananas comosus*		Johor, Malaysia	Mr. Ali Madu Nenas
**14**	NH	*Acacia mangium*		Malacca, Malaysia	Pak long MaduTani
**15**	OH	Multifloral		Malacca, Malaysia	
**16**	PH	Multifloral	*Apis trigona*	Malacca, Malaysia	
**17**	QH	Multifloral	*Apis mellifera*	Malacca, Malaysia	Giant B (Mr.Ong)
**18**	RH	Multifloral	*Apis trigona*	Malacca, Malaysia	
**19**	SH	*Acacia mangium*	*Apis mellifera*	Sarawak, Malaysia	Summer Pacific (Mr.Esa)
			**NEW ZEALAND**		
**20**	ANZ	Manuka (< Clover, Honey dew)	*Apis mellifera*	Wye Valley, New Zealand	The Honey Company Ltd (Natasha)
**21**	BNZ	Manuka (< Borage, Clover)		Upper Tanaka, New Zealand	
**22**	CNZ	Manuka (< Borage, Clover)		Upper Wairau, New Zealand	
**23**	DNZ	Manuka (< Borage, Clover)		Wairau, New Zealand	
**24**	ENZ	Manuka		Linkwater, New Zealand	
**25**	FNZ	Manuka		Keneperu, New Zealand	
**26**	GNZ	Manuka (< Borage, Clover, Matagouri)		Moutere	
**27**	HNZ	Manuka (< Borage, Clover, Kamahi)		Wairau Valley, New Zealand	
**28**	INZ	Manuka (< Clover)		Awatere Valley, New Zealand	

**29**	JNZ	Manuka (< Clover, Honey dew)	Awatere Valley, New Zealand	
**30**	KNZ	Manuka /Manuka blend	New Zealand	Honey New Zealand (International) Ltd (Dr. Young)
**31**	LNZ	*Knightia excels* (Rewarewa)	New Zealand	
**32**	MNZ	Manuka Manuka blend	New Zealand	
**33**	NNZ	Manuka Manuka blend	New Zealand	
**34**	ONZ	Manuka Manuka blend	New Zealand	
**35**	PNZ	Manuka Manuka blend	New Zealand	
**36**	QNZ	Manuka Manuka blend	New Zealand	
**37**	RNZ	Manuka Manuka blend	New Zealand	
**38**	SNZ	Manuka Manuka blend	New Zealand	
**39**	TNZ	Manuka Manuka blend	New Zealand	
**40**	UNZ	Manuka Manuka blend	New Zealand	
**41**	VNZ	Manuka Manuka blend	New Zealand	
**42**	WNZ	Multifloral (Wild floral)	New Zealand	

References

Boffo, E. F., Tavares, L. A., Tobias, A. C. T., Ferreira, M. M. C., & Ferreira, A. G. (2012). Identification of components of Brazilian honey by 1H NMR and classification of its botanical origin by chemometric methods. *LWT-Food Science and Technology, 49*(1), 55-63.

Lee, R., Harris, J., Champagne, P., & Jessop, P. G. (2016). CO 2-Catalysed conversion of carbohydrates to 5-hydroxymethyl furfural. *Green Chemistry, 18*(23), 6305-6310.

Macintyre, L.; Zhang, T.; Viegelmann, C.; Juarez Martinez, I.; Cheng, C.; Dowdells, C.; Abdelmohsen, U.R.; Gernert, C.; Hentschel, U.; Edrada-Ebel, R. Metabolomic tools for secondary metabolite discovery from marine microbial symbionts. *Mar. Drugs* **2014**, *12*, 3416–3448. https://doi.org/10.3390/md12063416.

Ohmenhaeuser, M., Monakhova, Y. B., Kuballa, T., & Lachenmeier, D. W. (2013). Qualitative and quantitative control of honeys using NMR spectroscopy and chemometrics. *ISRN Analytical Chemistry, 2013*.

Spiteri, M., Jamin, E., Thomas, F., Rebours, A., Lees, M., Rogers, K. M., & Rutledge, D. N. (2015). Fast and global authenticity screening of honey using 1H-NMR profiling. *Food Chemistry, 189*, 60-66.
